# Bis(trimethylsilyl)phosphide chemistry: a half-century of advances across the periodic table

**DOI:** 10.1039/d4cs01141d

**Published:** 2025-03-12

**Authors:** Jack Baldwin, David P. Mills

**Affiliations:** a Department of Chemistry, The University of Manchester Oxford Road Manchester M13 9PL UK david.mills@manchester.ac.uk

## Abstract

Whilst bis(trimethylsilyl)amide has been used extensively as a ligand across the periodic table, the chemistry of its heavier group 15 congeners is relatively underdeveloped. However, bis(trimethylsilyl)phosphide coordination chemistry has provided unique structural motifs and has also shown potential applications in catalysis, materials science, and bioinorganic chemistry. This review, which marks 55 years since the first report of a bis(trimethylsilyl)phosphide complex, provides a comprehensive overview of the synthesis, characterisation and reactivity of structurally authenticated s-, p-, d- and f-block metal complexes of this ligand, focusing on salient single crystal XRD and NMR spectroscopic data. We discuss the factors influencing the diverse coordination modes and reactivity profiles of bis(trimethylsilyl)phosphide complexes, together with an overview of their potential as precursors for novel solid-state materials, aiming to inspire future research endeavours using this ligand. We also review the small number of bis(triisopropylsilyl)phosphide complexes, in order to provide motivation for the future study of other bis(silyl)phosphide ligands.

## Abstract 2

We review structurally authenticated metal(loid) bis(trimethylsilyl)phosphide complexes, highlighting how their solid-state structures and electronic structures differ from those containing the related bis(trimethylsilyl)amide ligand.

## Graphical abstract

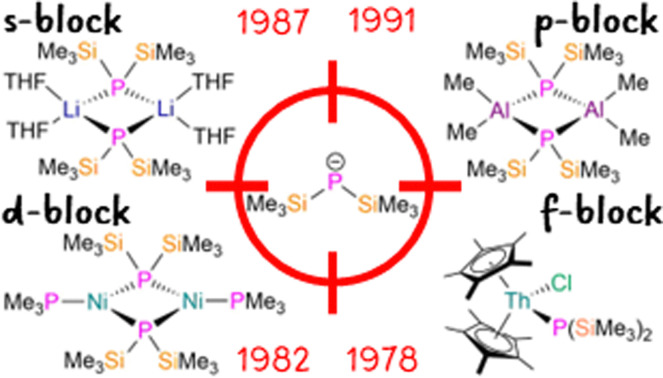

## 1. Introduction

Bis(trimethylsilyl)amide, {N(SiMe_3_)_2_} (N′′), and derivatives thereof, have been used extensively across the periodic table to provide landmark complexes^1–7^ since the first s-block examples were reported by Wannagat in 1961.^8^ The popularity of N′′ in coordination chemistry can be attributed to a combination of: (i) kinetic protection of the metal coordination sphere and inter-ligand dispersion force stabilisation provided by sterically demanding SiMe_3_ groups; (ii) the absence of potential β-hydride elimination decomposition pathways; (iii) the high lipophilicity of SiMe_3_ groups increasing complex solubility in non-polar solvents and facilitating crystal growth for structure determination; (iv) negative hyperconjugation by the silyl groups making the ligand charge more diffuse, resulting in softer N-donor atoms and promoting additional stabilising M⋯Si–C and M⋯C–H interactions; (v) NMR spectra that are typically easy to interpret; and, (vi) the ease of preparation and commercial availability of s-block ligand transfer agents.^1–7^

**Fig. 1:**
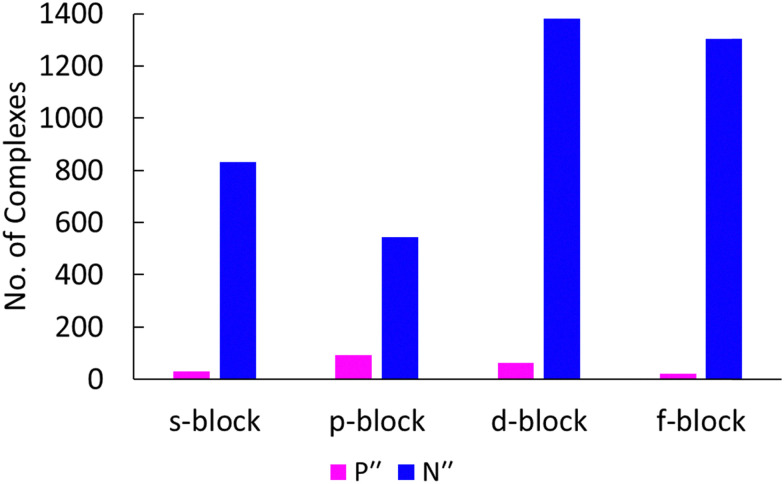
Graph depicting the number of structurally authenticated N′′ *vs*. P′′ metal complexes containing elements from each block of the periodic table.^9^

**Scheme 1:**
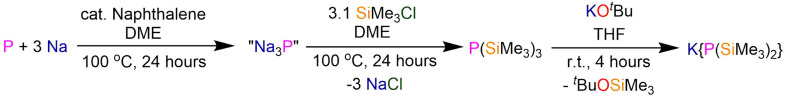
Synthesis of KP′′ from red phosphorus, sodium and trimethylsilylchloride.^10–13^

The coordination chemistry of bis(trimethylsilyl)phosphide, {P(SiMe_3_)_2_} (P′′), is immature in comparison to that of the lighter congener N′′, despite it having many of the same advantages. This is evident from a survey of the Cambridge Structural Database (CSD), which shows 210 entries for P′′ *vs*. 4062 entries for N′′ structurally authenticated metal complexes (non-metals and the metalloids B, Si, As, Sb and Te were excluded from this search and are outside the scope of this review); the 210 P′′ metal complexes consist of 94 p-, 64 d-, 30 s- and 22 f-block examples (Fig. 1).^9^ This can be ascribed to difficulties in handling s-block P′′ salts, which are pyrophoric, malodorous and toxic, and the synthesis of these ligand transfer agents typically using P(SiMe_3_)_3_ as a starting material (Scheme 1).^10–13^ As well as having similar handling concerns to P′′, P(SiMe_3_)_3_ is relatively expensive, and thus is typically synthesised from red phosphorus, sodium and chlorotrimethylsilane on large scales in dimethoxyethane (DME) (Scheme 1); this synthetic procedure can discourage investigations as it is inherently hazardous.^14^ However, an advantage of P′′ over N′′ is that the 100% abundant *I* = ½ metal-bound ^31^P nuclei can provide a useful spectroscopic handle, *e.g.* by using NMR or EPR spectroscopy. P′′ complexes could also potentially deliver unique applications as P′′ is softer than N′′ and has a higher propensity to bridge metal ions as M–P bonds are longer than M–N bonds.^15–17^ Since the first reported syntheses of s-block P′′ complexes^18^ they have been used in a plethora of reactions, ranging from nucleophilic additions to radical-mediated processes;^19^ they have also been shown to be versatile reducing agents,^20^ and a precursor to phosphorus-centred radicals.^19^

Here we provide a review of P′′ coordination chemistry, focusing on structurally authenticated examples of complexes containing direct M–P bonds. When appropriate we make comparisons to complexes of the lighter congener, N′′; previous reviews of metal phosphido chemistry have tended to cover a wider range of ligand substituents and a narrower range of elements.^21–23^ The review is divided into sections of s-, p-, d-, and f-block metal P′′ complexes; these sections are subdivided by group number, with heterometallic ‘ate’ complexes containing s-block elements included in the p-, d- or f-block sections as appropriate. Where M–P bond lengths from single crystal XRD studies and chemical shifts and coupling constants from ^31^P and ^29^Si (4.7% abundant, *I* = ½) NMR spectroscopic data have been reported they are compiled in Table 1 (calculation of the standard error of the mean (SEM) = SD/√N, where SD = standard deviation and *N* = sample size). We focus on discussions of these data in this review, which we intend to provide a useful resource to the community and to inspire future research in P′′ coordination chemistry. We also provide coverage of nascent bis(triisopropylsilyl)phosphide chemistry, in order to show that other bis(silyl)phosphide ligands should provide new avenues to explore in future.

**Table 1:** Mean M–P bond lengths (Å), and chemical shifts (*δ*) and coupling constants (Hz) for ^31^P and ^29^Si NMR spectroscopic data, of structurally authenticated P′′ complexes^9^

Complex	Mean M–P (Å)	Solvent	^31^P Chemical shift (*δ*)	^31^P Coupling constant (Hz)	^29^Si Chemical shift (*δ*)	^29^Si Coupling constant (Hz)	Ref.
s-Block	2.62(2)	*d*_8_-Toluene	−297.6, s	—	—	—	24
1 [Li(μ-P′′)(THF)_2_]_2_							
2 [Li(μ-P′′)(DME)]_2_	2.559(4)	—	—	—	—	—	25
3 [Li_4_(μ_2_-P′′)_2_(μ_3_-P′′)_2_(THF)_2_]	2.50(4)^a^	*d*_8_-Toluene	−297.7, s	—	—	—	24
	2.56(4)^b^						
4 [Li_6_(μ_2_-P′′)_2_(μ_3_-P′′)_4_]	2.51(1)^a^	—	—	—	—	—	26
	2.517(16)^b^						
5-K [K_4_(μ_2_-P′′)_2_(μ_3_-P′′)_2_]_∞_	3.4168(11)^a^	THF	−293.4, s	—	0.70, s	^1^ *J*_PSi_ = 54	27
	3.171(6)^b^						
5-Rb [Rb_4_(μ_2_-P′′)_2_(μ_3_-P′′)_2_]_∞_	3.485(2)^a^	THF	−287.1, s	—	0.41, s	^1^ *J*_PSi_ = 55	27
	3.4157(12)^b^						
5-Cs [Cs_4_(μ_2_-P′′)_2_(μ_3_-P′′)_2_]_∞_	3.656(2)^a^	THF	−270.0, s	—	0.50, s	^1^ *J*_PSi_ = 52	27
	3.5816(12)^b^						
6 [Cs(μ-P′′)(μ-1,4-dioxane)_3_(1,4-dioxane)]_∞_	3.6137(17)	*d*_8_-THF	−276.1, s	—	—	—	28
7 [Mg(μ-P′′)(Br)(THF)]_2_	2.5624(16)	*d*_6_-Benzene	−296.9, br	—	2.9, br	—	20
8 [Mg_3_(μ-P′′)_4_(P′′)_2_]	2.594(7)^c^	*d*_8_-Toluene	−242.55, t^c^	^2^ *J*_PP_ = 17.9	3.74, s	—	29
	2.455(4)^d^		−275.44, t^d^				
9 [Mg{P′′C(Ph)C(C_2_Ph)}(μ-P′′)]_2_	2.5576(19)^c^	*d*_8_-Toluene	−262.29, t^c^	^2^ *J*_PP_ = 5	4.56, s^c^	^1^ *J*_PSi_ = <2^c^	30
	2.6998(17)		−98.4, t		3.63, d	^1^ *J*_PSi_ = 6.3	
10 [Mg(P′′)_2_(DME)]	2.487(2)	—	—	—	—	—	31
11 [Mg(P′′)_2_(THF)_2_]	2.5023(19)	*d*_8_-Toluene	−294.7, s	—	1.81, s	^1^ *J*_PSi_ = 33.0	32
12-Ca [Ca(N′′)(μ-P′′)(sol)]_2_	2.9269(18)	*d*_6_-Benzene	−229.96, s^e^	—	5.42, d^e^	^1^ *J*_PSi_ = 18.7^e^	33
			−254.82, s^f^	—	−1.77, d^f^	^1^ *J*_PSi_ = 22.0^f^	
12-Ba [Ba(N′′)(μ-P′′)(sol)]_2_	3.289(2)	*d*_8_-THF	−222.05, s	—	1.03, s	^1^ *J*_PSi_ = 34.8	34
13-Ca*trans*-[Ca(P′′)_2_(THF)_4_]	2.917(4)	—	—	—	—	—	33
13-Sr*trans*-[Sr(P′′)_2_(THF)_4_]	3.021(10)	*d*_8_-Toluene	−256.2, s	—	1.11, s	^1^ *J*_PSi_ = 34.1	35
13-Ba*trans*-[Ba(P′′)_2_(THF)_4_]	3.175(10)	*d*_8_-THF	−251.0, s	^2^ *J*_PP_ = 23.5	1.08, s	^1^ *J*_PSi_ = 44.5	36
						^3^ *J*_PSi_ = 1.0	
14 [Sr(P′′)(μ-P′′)_3_Sr(THF)_3_]	3.118(8)^c^	*d*_8_-Toluene	−276.78, s^d^	^2^ *J*_PP_ = 22.5^d^	1.60, s	^1^ *J*_PSi_ = 38.4^d^	35
	3.042(4)^d^						
15 [Ca(P′′)_2_(TMTA)_2_]	2.995(2)	*d*_8_-THF	−276.8, s^d^	^2^ *J*_PP_ = 14.5	0.87, s	^1^ *J*_PSi_ = 34.4	36
						^3^ *J*_PSi_ = −2.7	

p-Block							
16 [Al(P′′)(tmp)_2_]	2.361(2)	*d*_6_-Benzene	−238.00, s	—	—	—	37
17-AlMe [Al(Me)_2_(P′′)(dmap)]	2.3768(9)	*d*_6_-Benzene	—	—	—	—	38
17-GaMe [Ga(Me)_2_(P′′)(dmap)]	2.372(1)	*d*_8_-Toluene	−273.80, s	—	—	—	39
17-Ga^*t*^Bu [Ga(^*t*^Bu)_2_(P′′)(dmap)]	2.3948(6)	*d*_6_-Benzene	−281.00, s	—	—	—	40
18 [Ga(P′′)I(^*t*^Bu-DAB)]	2.2991(11)	—	—	—	—	—	41
19 [Ga(P′′)_2_(^*t*^Bu-DAB)]	2.3426(18)	—	—	—	—	—	41
20-Ga [Ga(^Dipp^Nacnac)(P′′)(Cl)]	2.3310(9)	*d*_6_-Benzene	−255.00, s	—	—	—	42
20-In [In(^Dipp^Nacnac)(P′′)(Cl)]	2.4806(8)	*d*_6_-Benzene	−252.90, s	—	—	—	42
21-AlMe [Al(Me)_2_(μ-P′′)]_2_	2.457(2)	*d*_8_-Toluene	−226.7, s	—	6.24, Vir. t	^1^ *J*_PSi_ = 9.9	43 and 44
21-AlEt [Al(Et)_2_(μ-P′′)]_2_	2.458(1)	*d*_6_-Benzene	−246.90, s	—	—	—	45
21-AlCH_2_^i^Pr [Al(CH_2_^i^Pr)_2_(μ-P′′)]_2_	2.476(2)	*d*_6_-Benzene	−245.30, s	—	—	—	43
21-AlCH_2_SiMe_3_ [Al(CH_2_SiMe_3_)_2_(μ-P′′)]_2_	2.483(1)	*d*_8_-Toluene	−231.49, s	—	6.38, s	^1^ *J*_PSi_ = 6.2	46
21-GaMe [Ga(Me)_2_(μ-P′′)]_2_	2.450(1)	*d*_6_-Benzene	−219.20, s	—	6.41, s	—	40 and 47
21-GaEt [Ga(Et)_2_(μ-P′′)]_2_	2.4558(7)	*d*_8_-Toluene	−227.80, s	—	—	—	48
21-Ga^*n*^Bu [Ga(^*n*^Bu)_2_(μ-P′′)]_2_	2.4533(6)	*d*_6_-Benzene	−227.00, s	—	—	—	49
21-GaCH_2_^*t*^Bu [Ga(CH_2_^*t*^Bu)_2_(μ-P′′)]_2_	2.517(3)	*d*_6_-Benzene	−215.24, s	—	—	—	50
21-GaCH_2_SiMe_3_ [Ga(CH_2_SiMe_3_)_2_(μ-P′′)]_2_	2.4887(16)	*d*_6_-Benzene	−205.88, s	—	—	—	51
21-InMe [In(Me)_2_(μ-P′′)]_2_	2.630(1)	*d*_8_-Toluene	−239.80, s	—	—	—	52
21-InEt [In(Et)_2_(μ-P′′)]_2_	2.645(1)	*d*_6_-Benzene	−242.90, s	—	—	—	53
21-InPh [In(Ph)_2_(μ-P′′)]_2_	2.612(1)	*d*_6_-Benzene	−221.59, s	—	—	—	54
21-InCH_2_Ph [In(CH_2_Ph)_2_(μ-P′′)]_2_	2.6123(6)	*d*_2_-DCM	−220.30, s	—	—	—	55
21-InCH_2_SiMe_3_ [In(CH_2_SiMe_3_)_2_(μ-P′′)]_2_	2.655(3)	*d*_6_-Benzene	−227.36, s	—	—	—	56
21-TlMe [Tl(Me)_2_(μ-P′′)]_2_	2.692(3)	*d*_6_-Benzene	−234.00, s	^1^ *J*_TlP_ = 2462	—	—	57
22 [Al(Me)_2_(dmap)(μ-P′′)Ga(Me)_3_]	2.416(1)	*d*_8_-Toluene	−262.40, s	—	—	—	58
23-CH_2_SiMe_3_ [In(Me)(CH_2_SiMe_3_)(μ-P′′)]_2_	2.635(2)	*d*_6_-Benzene	−234.32, s^g^	—	—	—	59
			−234.67, s^h^				
23-CH_2_^*t*^Bu [In(Me)(CH_2_^*t*^Bu)(μ-P′′)]_2_	2.637(3)	*d*_6_-Benzene	−239.56, s^g^	—	—	—	60
			−239.42, s^h^				
24 [{In(CH_2_^*t*^Bu)_2_}_2_{μ-P′′}{μ-PH(SiMe_3_)}]	2.650(5)	*d*_6_-Benzene	−209.92, s	^1^ *J*_PH_ = 473	—	—	60
			−209.12, s				
25-AlBrCH_2_SiMe_3_ [Al(CH_2_SiMe_3_)(Br)(μ-P′′)]_2_	2.436(4)	*d*_6_-Benzene	−215.15, s	—	—	—	61
25-GaClMe [Ga(Me)(Cl)(μ-P′′)]_2_	2.4106(10)	—	—	—	—	—	62
25-GaClCH_2_^*t*^Bu [Ga(Cl)(CH_2_^*t*^Bu)(μ-P′′)]_2_	2.422(3)	*d*_6_-Benzene	−233.86, s	—	—	—	50
25-GaBrCH_2_SiMe_3_ [Ga(CH_2_SiMe_3_)(Br)(μ-P′′)]_2_	2.424(3)	*d*_6_-Benzene	−227.61, s	—	—	—	50
25-InClCH_2_SiMe_3_ [In(CH_2_SiMe_3_)(Cl)(μ-P′′)]_2_	2.593(2)	*d*_6_-Benzene	−241.43, s	—	—	—	59
25-InClCp* [In(Cp*)(Cl)(μ-P′′)]_2_	2.621(2)	*d*_6_-Benzene	−148.60, s	—	—	—	63
26-GaPh [{Ga(Ph)_2_}_2_(μ-P′′)(μ-Cl)]	2.391(4)	*d*_6_-Benzene	−214.74, s	—	—	—	64
26-GaCH_2_^*t*^Bu [{Ga(CH_2_^*t*^Bu)_2_}_2_(μ-P′′)(μ-Cl)]	2.451(3)	*d*_6_-Benzene	−210.22, s	—	—	—	50
26-GaCH_2_SiMe_3_ [{Ga(CH_2_SiMe_3_)_2_}_2_(μ-P′′)(μ-Cl)]	2.416(6)	*d*_6_-Benzene	−213.27, s	—	—	—	50
26-InCH_2_^*t*^Bu [{In(CH_2_^*t*^Bu)_2_}_2_(μ-P′′)(μ-Cl)]	2.622(5)	*d*_6_-Benzene	−227.34, s	—	—	—	60
26-InCH_2_SiMe_3_ [{In(CH_2_SiMe_3_)_2_}_2_(μ-P′′)(μ-Cl)]	2.603(4)	*d*_6_-Benzene	−218.99, s	—	—	—	56
27-Al [{Al(Et)_2_}_2_(μ-P′′)(μ-As′′)]	2.497(1)	*d*_6_-Benzene	−240.93, s	—	—	—	65
			−245.83, s				
27-In [{In(CH_2_SiMe_3_)_2_}_2_(μ-P′′)(μ-As′′)]	2.691(3)	*d*_8_-Toluene	−229.84, s	—	—	—	66
			−230.72, s				
29 [{Ga(Et)_2_}_2_(μ-P′′)(μ-Sb′′)]	2.574(2)	*d*_8_-Toluene	—	—	—	—	48
30-ClP [{Ga(Cl)_2_}_2_(μ-P′′)(μ-P′′)]	2.379(3)	*d*_8_-Toluene	−158.40, s	—	—	—	67
30-BrP [{Ga(Br)_2_}_2_(μ-P′′)(μ-P′′)]	2.386(3)	*d*_8_-Toluene	−158.40, s	—	—	—	68
30-IP [{Ga(i)_2_}_2_(μ-P′′)(μ-P′′)]	2.398(4)	*d*_8_-Toluene	−158.40, s	—	—	—	68 and 69
30-IAs [{Ga(i)_2_}_2_(μ-P′′)(μ-As′′)]	2.443(3)	*d*_6_-Benzene	−261.25, s	—	—	—	69
31-Al [Al(H)_2_(μ-P′′)]_3_	2.398(2)	*d*_8_-Toluene	−273.50, s	—	—	—	70
31-Ga [Ga(H)_2_(μ-P′′)]_3_	2.392(3)	*d*_8_-Toluene	−265.80, s	—	—	—	71
32-Al [{Al(Me)_2_}_3_(μ-P′′){HP(SiMe_3_)}_2_]	2.4287(13)	*d*_8_-Toluene	−253.00, d^g^	[^1^*J*_PSi_ + ^2^*J*_PP_ = 85.0]^g^	—	—	72
			−252.46, d^h^	[^1^*J*_PSi_ + ^2^*J*_PP_ = 91.1]^h^			
32-Ga [{Ga(Me)_2_}_3_(μ-P′′){HP(SiMe_3_)}_2_]	2.415(2)	*d*_8_-Toluene	−241.74, t^g^	^2^ *J*_PP_ = 89.2^g^	5.53, dt^h^	^1^ *J*_PSi_ = 8.2^h^	72
			−241.01, t^h^	^2^ *J*_PP_ = 44.0^g^	6.0, dVir.t^h^	^3^ *J*_PSi_ = 6.9^h^	
				^2^ *J*_PP_ = 84.7^h^		[^1^*J*_PSi_ + ^3^*J*_PSi_ = 7.2]^h^	
				^2^ *J*_PP_ = 42.3^h^			
33 [{Ga(Me)_2_(μ-P′′)}_2_{μ-P(Me)}]_2_	2.443(9)	*d*_3_-Chloroform	−108.7, t	^2^ *J*_PP_ = 30.5	—	—	73
			−238.2, t				
34-Al [Al(H)_2_(μ-P′′)_2_Li(Et_2_O)_2_]	2.4001(13)	*d*_8_-Toluene	−282.0, br, s	—	—	—	74
34-Ga [Ga(H)_2_(μ-P′′)_2_Li(Et_2_O)_2_]	2.4122(12)	*d*_8_-Toluene	−277.7, s	—	—	—	75
35 [Al(H)_2_(μ-P′′)_2_Na(DME)_2_]	2.405(1)	*d*_6_-Benzene	−283.0, s	—	3.7, t	^1^ *J*_PSi_ = 9.8	76
36 [Ge(P′′)(Ar^Mes^)]	2.329(1)	*d*_6_-Benzene	−18.2, s	—	—	—	77
37-Ge [Ge(^Dipp^Nacnac)(P′′)]	2.3912(8)	*d*_6_-Benzene	−192.7, s	—	2.0, d	^1^ *J*_PSi_ = 17.1	78 and 79
37-Sn [Sn(^Dipp^Nacnac)(P′′)]	2.5526(7)	*d*_6_-Benzene	−183.5, s	^1^ *J*_PSi_ = 17.1	4.0, d	^1^ *J*_PSi_ = 17	78
				^1^ *J*_SnP_ = 1453			
37-Pb [Pb(^Dipp^Nacnac)(P′′)]	2.715(2)	*d*_6_-Benzene	−116.6, s	^1^ *J*_PbP_ = 2852	7.4, d	^1^ *J*_PSi_ = 36.0	78 and 80
38 [Ge(Se)(^Dipp^Nacnac)(P′′)]	2.2976(7)	*d*_6_-Benzene	−172.6, s	^1^ *J*_PSi_ = 26.0	—	—	81
				^1^ *J*_SeP_ = 52.0			
39-Ge [Ge(Ph*)(P′′)]	2.291(4)	*d*_6_-Benzene	−48.6, s	—	—	—	82
39-Sn [Sn(Ph*)(P′′)]	2.527(1)	*d*_6_-Benzene	−123.1, s	^1^ *J*_117SnP_ = 1396	4.08, d	^1^ *J*_PSi_ = 38.5	82
				^1^ *J*_119SnP_ = 1453			
40 [Pb(P′′)(μ-P′′)]_2_	2.77(1)^c^	*d*_6_-Benzene	−218.0, s^d^^g^	^1^ *J*_PbP_ = 1264^d^^g^	—	—	83
	2.70(1)^d^		−217.3, s^d^^h^	^1^ *J*_PbP_ = 1183^d^^h^			
			−281.4, s^c^^g^	^1^ *J*_PbP_ = 1658^c^^g^			
			−302.4, s^c^^h^	^1^ *J*_PbP_ = 1598^c^^h^			
41 [Sn(P′′)(μ-P′′)_2_Ca(μ-P′′)_2_Ca(N′′)]	Sn–P: 2.695(4)^c^	*d*_6_-Benzene	−220.0, br, s (SnP)	—	1.75, s (SnP*Si*_2_)	—	84
	2.795(9)^d^		−232.97, s (CaP)		5.70, s (CaP*Si*_2_)		
	Ca–P: 2.892(7)^c^						
42 [{Sn(P′′)(μ_2_-P′′)_2_}_2_Ba]	Sn–P: 2.684(7)^c^	*d*_8_-Toluene	−235.35, s	^1^ *J*_SnP_ = 1068.13	4.5, s	—	34
	2.600(6)^d^						
	Ba–P: 3.239(7)^c^						
43-Ca [Ca(THF)_2_{Sn(μ_2_-P′′)(μ_3_-P′)}_2_]	Ca–P: 2.903(2)	*d*_6_-Benzene	−223.58, s	^1^ *J*_SnP_ = 710.7	—	—	33
	Sn–P: 2.642(1)		(SnP)	^3^ *J*_SnP_ = 74.1			
			−295.15, s	(SnP)			
			(Sn_2_P)	^1^ *J*_SnP_ = 578.6			
				^2^ *J*_PP_ = 77.3			
				(SnP)			
43-Ba [Ba(THF)_*x*_{Sn(μ_2_-P′′)(μ_3_-P′)}_2_]	Ba–P: 3.302(12)	*d*_8_-Toluene	−220.30, s	^1^ *J*_SnP_ = 730.0	—	—	34
	Sn–P: 2.685(9)						

d-Block							
37-Cr [Cr(^Dipp^Nacnac)(P′′)]	2.3641(3)	—	—	—	—	—	85
37-Mn [Mn(^Dipp^Nacnac)(P′′)(THF)]	2.461(1)	—	—	—	—	—	85
37-Zn [Zn(^Dipp^Nacnac)(P′′)]	2.2728(3)	*d*_6_-Benzene	−288.85, s	^1^ *J*_PSi_ = 28.5	3.49, d	^1^ *J*_PSi_ = 28.5	85
44 [{Y(P′′)_2_}_2_(μ-P′′)_2_]	2.849(4)^c^	*d*_6_-Benzene	−107.80, dp^c^	^1^ *J*_YP_ = 56.7, ^2^*J*_PP_ = 5.0^c^	—	—	86 and 87
	2.678(4)^d^		−104.80, dt^d^	^1^ *J*_YP_ = 122.4, ^2^*J*_PP_ = 5.0^d^			
45 [Sc{C(PPh_2_S)_2_}(P′′)(py)_2_]	2.618(14)	*d*_8_-Toluene	−176.40, br, s	—	—	—	88
46-La [La{P(SiMe_3_)_2_}_3_(THF)_2_]	2.886(2)	*d*_6_-Benzene	−113.0, br	—	2.66, d	^1^ *J*_SiP_ = 22.4	89
47 [Ti(Cp)_2_(P′′)]	2.468(1)	—	251.0, br, s	—	—	—	90
48-Hf [Hf(Cp)_2_(P′′)_2_]	2.553(1)	*d*_6_-Benzene	−98.83, s	—	—	—	91
48-Zr [Zr(Cp)_2_(P′′)_2_]	—	*d*_6_-Benzene	−72.18, s	—	—	—	91
49-Hf [Hf(Cp)_2_(Cl)(P′′)]	—	*d*_6_-Benzene	−153.97, s	—	—	—	91
49-Zr [Zr(Cp)_2_(Cl)(P′′)]	2.547(6)	*d*_8_-Toluene	−108.90, s	—	—	—	92
50-Hf [Hf(Cp)_2_(Me)(P′′)]	—	*d*_6_-Benzene	−141.92, s	—	—	—	91
50-Zr [Zr(Cp)_2_(Me)(P′′)]	2.629(3)	—	—	—	—	—	91
51 [Zr(C_5_H_4_Me)_2_(P′′)_2_]	2.617(3)	*d*_6_-Benzene	−71.20, s	—	—	—	93
52 [Zr(Cp)_2_(Cl){P(SiMe_3_)_2_C(H)C(Ph)}]	2.855(4)	*d*_6_-Benzene	−179.50, s	—	—	—	94
53-Me [Zr(Cp)_2_(Me){P(SiMe_3_)_2_C(H)C(Ph)}]	2.915(3)	*d*_6_-Benzene	−179.30, s	—	—	—	94
53-^*n*^Bu [Zr(Cp)_2_(^*n*^Bu){P(SiMe_3_)_2_C(H)C(Ph)}]	—	*d*_6_-Benzene	−176.60, s	—	—	—	94
53-C≡CPh [Zr(Cp)_2_(C≡CPh){P(SiMe_3_)_2_C(H)C(Ph)}]	2.774(3)	*d*_6_-Benzene	−189.90, s	—	—	—	95
54-Cr [Cr(Cp)(μ-P′′)]_2_	2.3839(13)	*d*_6_-Benzene	No resonance observed	—	—	—	96
54-Mn [Mn(Cp)([μ-P′′)]_2_]	2.5099(7)	—	—	—	—	—	85
55 [Li(12-crown-4)_2_][{Mo(Cp)(CO)_2_}_2_(μ-P′′)]	2.4304(6)	—	—	—	—	—	97
56 [1,2-W_2_(P′′)(NMe_2_)_4_]	2.423(3)	*d*_6_-Benzene	−106.70, s^i^	^1^ *J*_WP_ = 243^i^	—	—	98
			−88.30, s^j^	^1^ *J*_WP_ = 250^j^			
	
57 [1,2-W_2_(PCy_2_)(P′′)(NMe_2_)_4_]	2.425(4)	*d*_6_-Benzene	−106.90, s^i^	^1^ *J*_WP_ = 239^i^	—	—	98
			−96.30, s^j^	^1^ *J*_WP_ = 234^j^			
58 [{Mn(P′′)(μ-P′′)_2_}{Mn(P′′)(THF)}]	2.526(5)^c^	—	—	—	—	—	82
	2.439(4)^d^						
59-EtMe_4_ [Fe(C_5_EtMe_4_)(CO)_2_(P′′)]	2.359(3)	*d*_6_-Benzene	−219.10, s	—	6.84, d	^1^ *J*_PSi_ = 46.7	99
59-^*n*^BuMe_4_ [Fe(C_5_^*n*^BuMe_4_)(CO)_2_(P′′)]	—	*d*_6_-Benzene	−218.60, s	—	6.84, d	^1^ *J*_PSi_ = 46.4	99
59-1,3-^*t*^Bu_2_H_3_ [Fe(C_5_H_3_^*n*^Bu_2_-1,3)(CO)_2_(P′′)]	—	*d*_6_-Benzene	−266.40, s	—	7.63, d	^1^ *J*_PSi_ = 53.0	99
60-Et [Ni(κ^2^-depe)(P′′)]	—	—	—	—	—	—	100
60-Cy [Ni(κ^2^-dcpe)(P′′)]	2.225(2)	—	—	—	—	—	100
60-Ph [Ni(κ^2^-dppe)(P′′)]	—	—	—	—	—	—	100
61-Ni^*n*^Bu [Ni(Br)(^*n*^Bu_2_-bimy)_2_(P′′)]	—	*d*_6_-Benzene	−197.30, s	—	—	—	101
61-Ni^i^Pr [Ni(i)(^i^Pr_2_-bimy)_2_(P′′)]	—	*d*_6_-Benzene	−178.60, s	—	—	—	101
61-Pd^*n*^Bu [Pd(i)(^*n*^Bu_2_-bimy)_2_(P′′)]	2.3648(17)	*d*_6_-Benzene	−192.80, s	—	—	—	101
61-Pd^i^Pr [Pd(i)(^i^Pr_2_-bimy)_2_(P′′)]	2.3442(12)	*d*_6_-Benzene	−181.70, s	—	—	—	101
62 [Ni(PMe_3_)(μ-P′′)]_2_	2.381(1)^c^	—	—	—	—	—	102
	2.129(2)^d^						
63-Pd [{Pd(PPh_3_)}_2_(P′′){Si[N(^*t*^Bu)]_2_CPh}]	—	*d*_8_-THF	−173.00, t	^2^ *J*_PP_ = 5.2	0.01, d	^1^ *J*_PSi_ = 6.0	103
63-Pt [{Pt(PPh_3_)}_2_(P′′){Si[N(^*t*^Bu)]_2_CPh}]	2.364(3)	*d*_6_-Benzene	−94.60, t	^2^ *J*_PP_ = 33.3	8.3, d	^1^ *J*_PSi_ = 7.4	103
				^1^ *J*_PSi_ = 219.2			
				^1^ *J*_PtP_ = 1633			
64 [Cu(^i^Pr_2_-bimy)_2_(P′′)]	2.291(7)	*d*_6_-Benzene	−261.00, s	—	—	—	104
65 [Au(^i^Pr_2_-bimy)(P′′)]	—	*d*_6_-Benzene	−235.00, s	—	—	—	105
66-Cu [Cu(^i^Pr)(P′′)]	2.1913(15)	*d*_6_-Benzene	−268.00, s	—	—	—	104
66-Au [Au(^i^Pr)(P′′)]	2.3174(10)	*d*_6_-Benzene	−235.70, s	—	—	—	105
67 [Au(CAAC^MeEt^)(P′′)]	2.3253(5)	*d*_6_-Benzene	−233.10, s	—	—	—	106
68-Cu [Cu(μ-P′′)]_6_	2.210(4)	*d*_6_-Benzene	−149.00, s	—	—	—	104
68-Ag [Ag(μ-P′′)]_6_	2.404(4)	*d*_6_-Benzene	−236.00, s	—	—	—	104
69 [Hg(P′′)_2_]	2.406(1)	*d*_6_-Benzene	−162.00, s	—	—	—	83
70-Zn [Zn(P′′)(μ-P′′)]_2_	2.420(1)^c^	*d*_6_-Benzene	−183.00, br, s^c^				
	2.295(1)^d^		−237.30, br, s^d^	—	—	—	83
70-Cd [Cd(P′′)(μ-P′′)]_2_	2.594(1)^c^	*d*_6_-Benzene	−180.10, br, s^c^	—	—	—	83
	2.459(1)^d^		−229.50, br, s^d^				
71-^*t*^Bu [Zn(^*t*^Bu)(μ-P′′)]_2_	—	*d*_6_-Benzene	−216.70, s^c^	—	4.17, s	—	107
71-^i^Pr [Zn(^i^Pr)(μ-P′′)]_2_	2.411(4)	*d*_6_-Benzene	−216.30, s	—	4.24, s	—	107
71-CH_2_SiMe_3_ [Zn(CH_2_SiMe_3_)(μ-P′′)]_2_	2.415(1)	*d*_6_-Benzene	−205.80, s	—	4.38, s	—	107
72-Zn [Zn(P^*n*^Pr_3_)Cl(μ-P′′)]_2_	2.437(1)^c^	—	—	—	—	—	108
	2.419(1)^d^						
72-Cd [Cd(P^*n*^Pr_3_)Br(μ-P′′)]_2_	2.590(1)^c^	—	—	—	—	—	108
	2.578(1)^d^						
73 [{Zn(Cl)(MeCN)(μ-P′′)_2_}_2_{Zn(μ-Cl)}_2_]	2.374(3)	—	—	—	—	—	108
74 [N^*n*^Bu_4_]_2_[Cd_4_(i)_8_(P′′)_2_]	2.507(7)	—	—	—	—	—	108
75-Me [Zn(Me)(P′′)]_3_	2.390(3)	*d*_6_-Benzene	−246.80, s	—	4.62, s	—	107
75-Et [Zn(Et)(P′′)]_3_	—	*d*_6_-Benzene	−246.90, s	—	4.61, s	—	107
75-^i^Pr [Zn(^i^Pr)(P′′)]_3_	2.408(6)	*d*_6_-Benzene	−243.60, s	—	—	—	107
75-^*n*^Bu [Zn(^*n*^Bu)(P′′)]_3_	2.388(4)	*d*_6_-Benzene	−246.00, s	—	4.67, s	—	107
76-Cr [Cr(CO)_5_(μ-P′′){Al(dmap)(Me_2_)}]	2.528(1)	*d*_2_-DCM	−278.10, s	—	—	—	39
76-Fe [Fe(CO)_4_(μ-P′′){Al(dmap)(Me_2_)}]	2.377(1)	*d*_8_-Toluene	−258.40, s	—	—	—	39
76-Ni [Ni(CO)_3_(μ-P′′){Al(dmap)(Me_2_)}]	2.315(2)	*d*_6_-Benzene	−275.20, s	—	—	—	39 and 109
77-Cr [Zr(C_5_H_4_Me)_2_(μ-P′′)_2_Cr(CO)_*n*_]	2.656(6)	*d*_6_-Benzene	−38.40, s	—	—	—	110
77-Mo [Zr(Cp)_2_(μ-P′′)_2_Mo(CO)_4_]	2.6648(11)	*d*_6_-Benzene	−57.90, s	—	—	—	110
77-Ni [Zr(Cp)_2_(μ-P′′)_2_Ni(CO)_2_]	2.654(1)	*d*_6_-Benzene	−42.10, s	—	—	—	111

f-Block							
46-Ce [Ce(P′′)_3_(THF)_2_]	2.849(3)	*d*_6_-Benzene	616.7, br	—	5.30, s	—	89
46-Pr [Pr(P′′)_3_(THF)_2_]	2.837(3)	*d*_6_-Benzene	1894.2, br	—	15.65, s	—	89
46-Nd [Nd(P′′)_3_(THF)_2_]	2.818(2)	*d*_6_-Benzene	2570.3, br	—	42.94, s	—	89 and 112
46-Sm [Sm(P′′)_3_(THF)_2_]	2.789(3)	*d*_6_-Benzene	−259.2, br	—	0.52, s	—	89
46-Tm [Tm(P′′)_3_(THF)_2_]	2.707(16)	*d*_6_-Benzene	—	—	—	—	113
78 [Sm(P′′)(μ-P′′)_3_Sm(THF)_3_]	3.039(5)^c^	*d*_8_-THF	—	—	—	—	114
	3.027(3)^d^						
79-Sm [{Sm(P′′)_3_(THF)}_2_(μ-I)K_3_(THF)]	3.033(5)	*d*_6_-Benzene	—	—	—	—	115
79-Eu [{Eu(P′′)_3_(THF)}_2_(μ-I)K_3_(THF)]	3.0316(4)	*d*_6_-Benzene	—	—	—	—	115
80 [KYb(P′′)_3_{μ-K(P′′)}_2_]_∞_	2.952(10)	*d*_6_-Benzene	—	—	—	—	115
81-Sm*trans*-[Sm(P′′)_2_(py)_4_]	3.0342(9)	*d*_6_-Benzene	—	—	—	—	115
81-Eu*trans*-[Eu(P′′)_2_(py)_4_]	3.0364(7)	*d*_6_-Benzene	—	—	—	—	115
81-Yb*trans*-[Yb(P′′)_2_(py)_4_]	2.928(13)	*d*_6_-Benzene	−253.93, s	^1^ *J*_YbP_ = 925	1.58, Vir. t	^1^ *J*_PSi_ = 16	115
82-Sm [Sm(P′′)_2_(18-crown-6)]	3.089(3)	*d*_6_-Benzene	—	—	—	—	115
82-Eu [Eu(P′′)_2_(18-crown-6)]	3.096(4)	*d*_6_-Benzene	—	—	—	—	115
82-Yb [Yb(P′′)_2_(18-crown-6)]	2.9662(10)	*d*_6_-Benzene	−265.58, s	^1^ *J*_YbP_ = 977	1.94, Vir. t	^1^ *J*_PSi_ = 17	115
83-Th [Th(P′′)(Cp*)_2_(Cl)]	—	*d*_6_-Benzene	109.0, s	—	—	—	116
83-U [U(P′′)(Cp*)_2_(Cl)]	2.788(4)	*d*_6_-Benzene	—	—	—	—	116
84-Th [Th(P′′)(Cp*)_2_(Me)]	2.888(4)	*d*_6_-Benzene	115.22, s	—	—	—	116
84-U [U(P′′)(Cp*)_2_(Me)]	2.893(4)	*d*_6_-Benzene	—	—	—	—	116
85-Th [Th{P(SiMe_3_)(SiMe_2_CH_2_)}(Cp*)_2_]	—	*d*_6_-Benzene	95.88, s	—	—	—	116
85-U [U{P(SiMe_3_)(SiMe_2_CH_2_)}(Cp*)_2_]	2.655(6)	*d*_6_-Benzene	—	—	—	—	116
86-Th [Th(Tren^DMBS^)(P′′)]	2.9406(11)	*d*_6_-Benzene	−100.09, s	—	—	—	117
86-U [U(Tren^DMBS^)(P′′)]	2.8646(14)	*d*_6_-Benzene	2055.21, s	—	—	—	117
87-Th [Th(Tren^TIPS^)(P′′)]	2.9020(13)	*d*_6_-Benzene	−66.45, s	—	8.49, s	—	117
87-U [U(Tren^TIPS^)(P′′)]	2.8391(9)	*d*_6_-Benzene	—	—	−97.09, s	—	117

a Refers to stile.

b Refers to rung bonds of ladder complexes.

c Refers to bridging.

d Refers to terminal P′′ ligands.

e Refers to *syn*-isomers.

f Refers to *anti*-isomers.

g Refers to *cis*-isomers.

h Refers to *trans*-isomers.

i Refers to the *anti*-conformation.

j Refers to the *gauche*-conformation.

### 2.1 s-Block P′′ complexes

Alkali metal and alkaline earth metal P′′ complexes are used extensively as ligand transfer agents, and therefore are important starting materials to p-, d- and f-block P′′ complexes. Surprisingly, to date there are no structurally characterised examples of Na P′′ complexes on the CSD,^9^ though various synthetic routes to group 1 P′′ salts including Na derivatives have been reported extensively.^11,12^ A Be P′′ complex has yet to be structurally authenticated to the best of our knowledge,^9^ thus there are obvious gaps in s-block P′′ chemistry that can be relatively easily addressed.

#### 2.1.1 Group 1 complexes

**Fig. 2:**
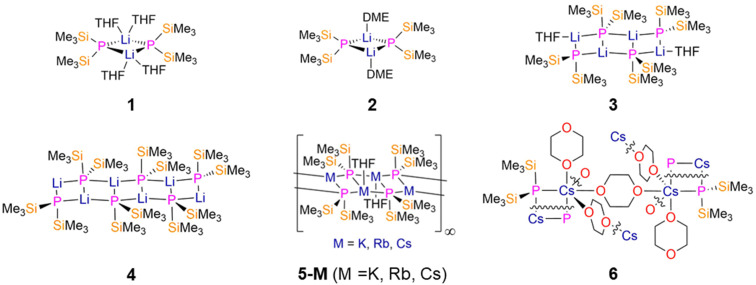
Group 1 M P′′ complexes [Li(μ-P′′)(THF)_2_]_2_ (1), [Li_4_(μ_2_-P′′)_2_(μ_3_-P′′)_2_(THF)_2_] (2), [Li(μ-P′′)(DME)]_2_ (3), [Li_6_(μ_2_-P′′)_2_(μ_3_-P′′)_4_] (4), [M_4_(μ_2_-P′′)_2_(μ_3_-P′′)_2_]_∞_ (5-M; M = K, Rb, Cs), [Cs(μ-P′′)(μ-1,4-dioxane)_3_(1,4-dioxane)]_∞_ (6).

To the best of our knowledge, the first report of a Li P′′ complex was by Bürger in 1970, but this was not structurally authenticated.^18^ The first isolated Li P′′ complex, [Li(P′′)(DME)], was reported in 1974 by Höldrich,^11,118^ but the first structurally characterised Li P′′ complexes were not published until 1987 by Rai.^24^ In Rai's report the reaction of *in situ*-formed P(SiMe_3_)_3_ with ^*n*^BuLi in THF was shown to give the dimeric complex [Li(μ-P′′)(THF)_2_]_2_ (1, Fig. 2); this undergoes oligomerisation when placed under vacuum at 20 °C for 20 hours to yield the ladder-like tetranuclear complex [Li_4_(μ_2_-P′′)_2_(μ_3_-P′′)_2_(THF)_2_] (3, Fig. 2).^24^ Similarly, the DME-solvated dimeric Li P′′ complex [Li(μ-P′′)(DME)]_2_ (2, Fig. 2) was later synthesised in 1989 by Becker from the reaction of P(SiMe_3_)_3_ with MeLi in DME.^25^ The solvent-free hexanuclear ladder-like complex, [Li_6_(μ_2_-P′′)_2_(μ_3_-P′′)_4_] (4, Fig. 2), was prepared in 1992 by Hey-Hawkins following treatment of HP′′ with ^*n*^BuLi in cyclopentane, showing how decreasing the amount of donor solvent molecules gives rise to an increase in aggregation.^26^ The mean Li–P bond lengths in 1 (2.62(2) Å) are longer than those found in 2 (2.559(4) Å) and the ladder complex 3 (2.50(4) Å for the Stiles and 2.56(4) Å for the rungs of the ladder).^24,25^ The six-step ladder complex 4 exhibits equal Li–P bond lengths for the Stiles of the ladder of 2.51(1) Å,^26^ but the Li–P distances of the rungs of the ladder vary considerably (2.38(1)–2.63(1) Å);^26^ the shortest Li–P bond lengths are seen for the μ_2_-P′′ at the termini as these Li ions are only two-coordinate, and unusually exhibit bent geometries.^119^ Complex 4 dissolves in THF and disaggregates to form complex 3.^26^ The related solvent-free Li N′′ complexes exist as cyclic trimers, [Li(μ-N′′)]_3_, in which the ring is planar,^120,121^ or as dimers in [Li(μ-N′′)]_2_.^122^

Similar ladder-like structures were obtained for P′′ complexes of the larger group 1 metals K, Rb and Cs by Ruhlandt-Senge and Uhlig in 1998.^27^ The polymeric heavy alkali metal P′′ complexes [M_4_(μ_2_-P′′)_2_(μ_3_-P′′)_2_]_∞_ (5-M; M = K, Rb, Cs, Fig. 2) were synthesised by the treatment of P(SiMe_3_)_3_ with the parent metal alkoxide MO^*t*^Bu (M = K, Rb, Cs) in THF, and were found to contain four-coordinate metal centres and five-coordinate phosphorus atoms in the solid state.^27^ Whilst complexes 5-K and 5-Rb were found to react rapidly with air and moisture, 5-Cs spontaneously ignites upon contact with air, owing to the increase in reactivity with the size of the alkali metal ion.^27^ A higher degree of oligomerisation was observed for the heavy group 1 P′′ complexes in the solid state in comparison to their lighter Li analogues;^26,27^ this is limited to growth of the Stiles of the ladder, signifying that the steric bulk of P′′ is sufficient to prevent oligomer growth in other directions. The M–P bond lengths of 5-M increase as the size of the metal increases from K-Cs; however, the K–P bond lengths of 5-K (3.3169(7), 3.4063(8), and 3.4273(8) Å) are slightly longer than expected from ionic radii trends. This was attributed to the steric demand of P′′ having a greater effect on the smaller K ion than for Rb or Cs, with 5-Rb and 5-Cs having mean M–P bond lengths of 3.486(2) Å and 3.656(2) Å, respectively.^27,123^

A 1,4-dioxane-bridged polymeric Cs P′′ complex [Cs(μ-P′′)(μ-1,4-dioxane)_3_(1,4-dioxane)]_∞_ (6, Fig. 2) was synthesised in 2008 by Ionkin by the reaction of P(SiMe_3_)_3_ with CsF in 1-4-dioxane at 90 °C for 3 hours.^28^ This study focussed on using CsF, P(SiMe_3_)_3_ and 2,4,6-tri-*tert*-butylphenylchloride (Mes*Cl) in order to form Mes*P′′, as this reaction does not occur at room temperature without reactive alkali metal precursors present.^28^ The reaction progress was monitored by ^31^P{^1^H} NMR spectroscopy as 6 shows a distinctive chemical shift of −276.10 ppm.^27,28^ As seen previously for the ladder-like oligomers 5, larger alkali metals like Cs give higher coordination numbers and higher order aggregates.^28^ The mean Cs–P bond length of 6 (3.6137(17) Å) is slightly longer than those seen for 5-Cs.^27,28^

#### 2.1.2 Group 2 complexes

**Fig. 3:**
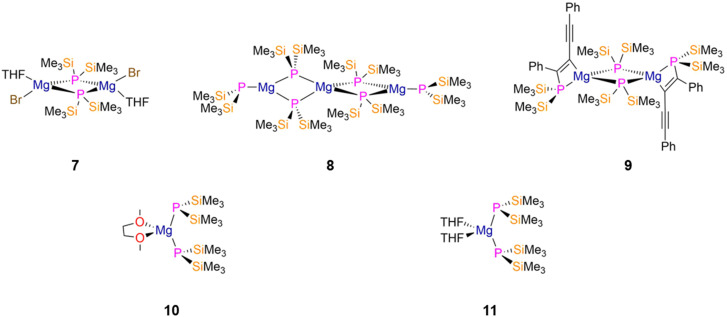
Mg P′′ complexes [Mg(μ-P′′)(Br)(THF)]_2_ (7), [Mg_3_(μ-P′′)_4_(P′′)_2_] (8), [Mg{P′′C(Ph)C(C_2_Ph)}(μ-P′′)]_2_ (9), [Mg(P′′)_2_(DME)] (10), [Mg(P′′)_2_(THF)_2_] (11).

Alkali metal ligand transfer agents often act as reducing agents and so are not always well-suited for straightforward salt metathesis reactions, therefore alkaline earth complexes such as Grignard reagents are often employed.^124^ The dimeric Mg P′′ complex [Mg(μ-P′′)(Br)(THF)]_2_ (7, Fig. 3) was prepared by Coles in 2010 *via* the protonolysis reaction of HP′′ with MeMgBr in Et_2_O.^20^ The related alkyl complex [Mg(μ-P′′)(Me)(THF)]_2_ was also identified in the reaction mixture by ^1^H NMR spectroscopy from the protonolysis reaction of HP′′ with MgMe_2_, which is present due to the Schlenk equilibrium of MeMgBr.^20^ A trinuclear Mg P′′ complex, [Mg_3_(μ-P′′)_4_(P′′)_2_] (8, Fig. 3), was synthesised by Westerhausen in 1998 by a similar protocol; in solution an equilibrium was observed with a dimeric form of this complex, formulated as [Mg_2_(μ-P′′)_2_(P′′)_2_], as evidenced by two sets of triplets in the ^31^P{^1^H} NMR spectrum (*δ*_P_ = −242.55 and −275.44 ppm; ^2^*J*_PP_ = 17.9 Hz) for the bridging and terminal P′′ ligands, respectively.^29^ A variable temperature (VT) experiment was performed in order to deconvolute the spectrum and show that the dinuclear complex is favoured at higher temperatures.^29^ The mean Mg–P_terminal_ bond lengths of the two periphery three-coordinate Mg ions in 8 (2.455(4) Å) are shorter than their mean Mg–P_bridging_ distances (2.546(5) Å), whilst the central four-coordinate Mg ion exhibits a range of Mg–P_bridging_ bond lengths (2.605(3)–2.678(2) Å). This is due to the large steric effect of four P′′ ligands about a single Mg ion in 8; the bridging Mg–P bond lengths in 7 (2.5624(7) Å) are shorter than those seen in 8.^20,29^

In 1998, Westerhausen reported the reaction of [Mg(P′′)_2_]_*n*_ with diphenylbutadiyne to give the dimeric [2 + 2]-cycloaddition product [Mg{P′′C(Ph)C(C_2_Ph)}(μ-P′′)]_2_ (9, Fig. 3), which contains MgPC_2_ four-membered rings with mean Mg–P_bridging_ bond lengths of 2.5576(19) Å.^30^ The formation of 9 was confirmed by ^31^P{^1^H} NMR spectroscopy, with two triplets at −98.4 and −262.3 ppm for the phosphine and bridging phosphide groups, respectively (^2^*J*_PP_ = 5 Hz).^30^ Evidence of weak conjugation of the triple and double bonds within 9 was provided by their respective stretching bands in the IR spectrum at 2131 and 1596 cm^−1^.^30^

The monomeric solvated Mg P′′ complexes [Mg(P′′)_2_(DME)] (10, Fig. 3) and [Mg(P′′)_2_(THF)_2_] (11, Fig. 3) were synthesised by Westerhausen in 1994 and 1995, respectively, by the separate protonolysis reactions of two equivalents of HP′′ with MgR_2_ (R = ^*n*^Bu, ^*sec*^Bu, N′′) in the respective donor solvent.^31,32^ Both complexes exhibit distorted tetrahedral geometries about the Mg ion, with the phosphorus atoms showing pyramidal geometries in the solid state with Mg–P bond lengths of 2.487(2) Å (10) and 2.5023(19) Å (11).^31,32^ Complex 11 exhibits a single resonance in the ^31^P{^1^H} NMR spectrum at −294.7 ppm; the respective NMR data for 10 were not reported but a similar chemical shift would be expected.^31,32^

**Fig. 4:**
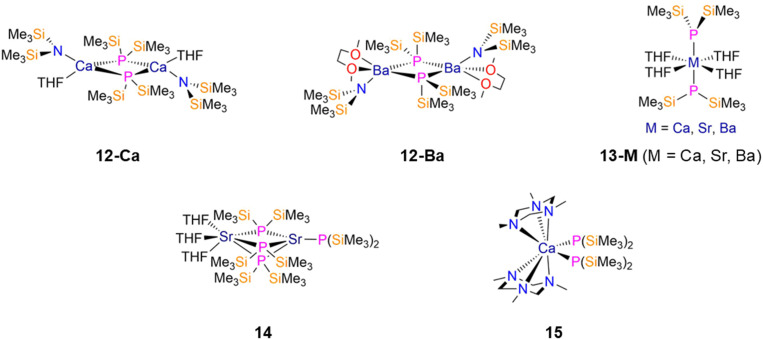
Heavy group 2 P′′ complexes [M(N′′)(μ-P′′)(sol)]_2_ (12-Ca: M = Ca, sol = THF; 12-Ba: M = Ba, sol = DME), *trans*-[M(P′′)_2_(THF)_4_] (13-M; M = Ca, Sr, Ba), [Sr(P′′)(μ-P′′)_3_Sr(THF)_3_] (14), [Ca(P′′)_2_(TMTA)_2_] (15, TMTA = 1,3,5-trimethyl-1,3,5-triazinane).

The dimeric heavy group 2 metal P′′ complexes, [M(N′′)(μ-P′′)(sol)]_2_ (12-Ca: M = Ca, sol = THF; 12-Ba: M = Ba, sol = DME; Fig. 4) were synthesised by Westerhausen in 1995 and 1996, respectively, by the separate reactions of [Ca(N′′)_2_(THF)_2_] or [Ba(N′′)_2_] with HP′′ in toluene or DME.^33,34^ These bimetallic complexes, which represented the first mixed bis(trimethylsilyl)pnictide complexes at the time, consist of two four-coordinate (12-Ca) or five-coordinate (12-Ba) metal centres, each with a bridging P′′, a terminal N′′, and monodentate THF for 12-Ca or bidentate DME for 12-Ba.^33,34^ The ^31^P{^1^H} NMR spectrum of 12-Ca indicates that a mixture of geometrical isomers is present in solution, with the *syn*-conformer resonance at (−229.96 ppm) and the *anti*-conformer at −254.82 ppm, whereas only one conformer is present in solution for 12-Ba (−222.05 ppm).^33,34^ For 12-Ca, it was noted that the central Ca_2_P_2_ ring is highly distorted, with Ca–P bond lengths of 2.927(2) and 3.005(2) Å, whereas the Ba_2_P_2_ ring in 12-Ba is essentially planar with Ba–P bond lengths of 3.289(2) and 3.340(2) Å; this is likely a consequence of the larger Ba ion better accommodating the bulky P′′ ligands.^33,34^

The protonolysis reaction of 12-Ca with HP′′ in THF gave the monomeric solvated P′′ complex *trans*-[Ca(P′′)_2_(THF)_4_] (13-Ca, Fig. 4).^33^ Similarly, the separate reactions of [M(N′′)_2_(THF)_*n*_] (M = Sr, *n* = 0; M = Ba, *n* = 2) with two equivalents of HP′′ in THF gave the heavier homologues, *trans*-[M(P′′)_2_(THF)_4_] (13-Ba,^36^13-Sr, Fig. 4).^35^ All three complexes exhibit distorted octahedral geometries with two axial P′′ and four equatorial THF, with M–P distances of 2.911(2) and 2.924(2) Å (13-Ca),^33^ 3.035(6) and 3.006(6) Å (13-Sr),^35^ and 3.158(6) and 3.190(6) Å (13-Ba),^36^ and near-linear P–M–P angles of 175.16(7)° (13-Ca),^33^ 174.2(2)° (13-Sr)^35^ and 174.9(1)° (13-Ba).^36^ When 13-Sr is heated under vacuum, the bimetallic complex, [Sr(P′′)(μ-P′′)_3_Sr(THF)_3_] (14, Fig. 4) forms, which has one four-coordinate Sr ion with three bridging and one terminal P′′, and one six-coordinate Sr ion with three bridging P′′ and three bound THF.^35^ Complex 14 is structurally analogous to that of the bimetallic Sm species discussed in Section 2.4.1.^125^

Finally, the reaction of [Ca(N′′)(μ-N′′)]_2_ with four equivalents each of HP′′ and 1,3,5-trimethyl-1,3,5-triazinane (TMTA) gave the eight-coordinate *C*_2_-symmetric Ca complex, [Ca(P′′)_2_(TMTA)_2_] (15, Fig. 4).^36^ This complex contains two P′′ and two tridentate TMTA ligands in chair conformations, with two identical Ca–P bond lengths of 2.994(2) Å, and a wide range of Ca–N bond lengths between 2.575(4)–2.704(5) Å, which was attributed to steric buttressing.^36^

### 2.2 p-Block complexes

As stated previously p-block P′′ compounds containing direct E–P bonds with E = non-metals and the metalloids B and Si are not within the scope of this review. Surprisingly no structurally authenticated examples of As, Sb, Bi or Te P′′ complexes were found in the CSD;^9^ we note that a synthetic route to a trimeric Bi(i) complex [Bi(μ-P′′)]_3_ has been reported and it has been claimed that the solid-state structure has been determined, but this dataset has not been deposited on the CSD.^12*e*^ The absence of group 15 and 16 metal(loid) P′′ complexes to date is noteworthy given that this chemistry has been developed for the lighter congener N′′.^7^ This section is therefore divided into group 13 and group 14 metal P′′ complexes, and subdivided into homo- and hetero-metallic examples, with the heavier metalloid Ge included for comparisons with heavier homologous Sn and Pb complexes.

#### 2.2.1 Homometallic Group 13 complexes

**Fig. 5:**
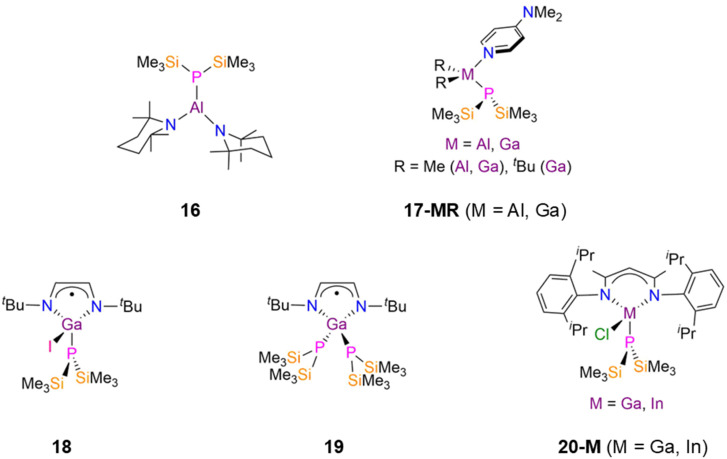
Monomeric group 13 P′′ complexes [Al(P′′)(tmp)_2_] (tmp = 2,2,6,6-tetramethyl-piperidino) (16), [M(R)_2_(P′′)(dmap)] (17-MR. 17-AlMe: M = Al, R = Me; 17-GaMe: M = Ga, R = Me; 17-Ga^*t*^Bu: M = Ga, R = ^*t*^Bu; dmap = 4-(dimethylamino)pyridine), [Ga(P′′)(i)(^*t*^Bu-DAB)], (18, ^*t*^Bu-DAB = {N(^*t*^Bu)C(H)}_2_), [Ga(P′′)_2_(^*t*^Bu-DAB)] (19), [M(^Dipp^Nacnac)(P′′)(Cl)] (^Dipp^Nacnac = {HC[C(Me)N(Dipp)]_2_}, Dipp = C_6_H_3_^i^Pr_2_-2,6) (20-M; M = Ga, In).

Group 13 P′′ complexes have received interest as reactive precursors for further chemical transformations such as in the formation of N-heterocyclic rings and cages.^37^ There are currently seven structurally characterised examples of mononuclear group 13 P′′ complexes, which all contain bulky supporting ligands.^37^ The Al(iii) P′′ complex [Al(P′′)(tmp)_2_] (tmp = 2,2,6,6-tetramethyl-piperidino) (16, Fig. 5) was synthesised by Nöth and Paine in 2007 by the salt metathesis reaction of [Al(tmp)_2_(Cl)] with LiP′′ in hexane.^37^ The reactions of CO_2_, CS_2_ and OCS with 16 proceeded *via* insertion into the Al–P bond to yield bridged species.^37^ From 2001–2006, Schulz reported synthetic routes to the four-coordinate M(iii) complexes [M(R)_2_(P′′)(dmap)] (17-MR. 17-AlMe: M = Al, R = Me; 17-GaMe: M = Ga, R = Me; 17-Ga^*t*^Bu: M = Ga, R = ^*t*^Bu; dmap = 4-(dimethylamino)pyridine, Fig. 5) which involved the addition of a base (dmap) to the heterocyclic complexes [M(R)_2_(P′′)]_*x*_ (*x* = 1–3).^38–40^ The M–P bond lengths of the methyl derivatives (17-AlMe: 2.375(1) Å; 17-GaMe: 2.372(1) Å) are relatively short, whilst that of 17-Ga^*t*^Bu is slightly longer (2.3948(6) Å) due to the additional steric effects of the ^*t*^Bu groups.^38–40^ The separate reactions of these complexes with various d-transition metal carbonyl complexes gave heterobimetallic complexes, which are discussed in Section 2.3.

In 2005, Jones and Murphy reported the synthesis of the Ga(iii) P′′ complexes, [Ga(P′′)(i)(^*t*^Bu-DAB)] (18, Fig. 5) and [Ga(P′′)_2_(^*t*^Bu-DAB)] (19, Fig. 5) (^*t*^Bu-DAB = {N(^*t*^Bu)C(H)}_2_), by the reaction of the Ga(ii) precursors [Ga(^*t*^Bu-DAB)(i)]_2_ with either two or four equivalents of MP′′ (M = Li, Na) in Et_2_O at −78 °C.^41^ The mechanism of formation of 18 is unclear, but involves both salt elimination and Ga–Ga bond cleavage, with oxidation of Ga(ii) to Ga(iii). When only one equivalent of MP′′ was used the unreacted Ga(ii) starting material was isolated, whilst the addition of a further equivalent of MP′′ to 18 gave 19 in moderate yield.^41^ Both of these Ga(iii) P′′ complexes have distorted tetrahedral geometries, with the Ga–P bond lengths being longer for 19 (2.343(2) Å mean) than for 18 (2.300(2) Å) due to the increased steric bulk about the metal centre.^41^ Complexes 18 and 19 did not give NMR data that could be interpreted due to their paramagnetism, but EPR spectroscopy showed that there was a small amount of unpaired spin density at the P atoms due to the splitting of the signal from coupling to ^31^P nuclei. A smaller line width was seen for 19 due to increased spin delocalisation at the P′′ ligands; despite this giving increased spin density at gallium, the unpaired electrons are mostly localised on the diazabutadiene ligands.^41^ In 2019, Scheer investigated a series of group 13 phosphido complexes for their potential applications in nanoparticles, semiconductors and optoelectronic layers.^42^ The authors postulated that bulky co-ligands would be needed in order to isolate mononuclear parent phosphido complexes, as there is a tendency towards oligomerisation due to the Lewis acidity of the group 13 metal centres and Lewis basicity of PH_2_.^42^ The reaction of [M(^Dipp^Nacnac)(Cl)_2_] (M = Ga, In; ^Dipp^Nacnac = {HC[C(Me)N(Dipp)]_2_}, Dipp = C_6_H_3_^i^Pr_2_-2,6) with excess LiP′′ in toluene gave the monosubstituted complexes [M(^Dipp^Nacnac)(P′′)(Cl)] (20-M; M = Ga, In, Fig. 5); the disubstituted P′′ complexes could not be prepared due to the steric bulk of the β-diketiminate ligand.^42^^31^P{^1^H} NMR spectroscopy revealed singlet resonances at −255.0 ppm (20-Ga) and −252.9 ppm (20-In), whilst single crystal XRD showed that these complexes have heavily distorted tetrahedral geometries, with N–M–N angles of 96.03(13)° (20-Ga) and 90.33(10)° (20-In); the M–P bond length of 2.3310(9) Å (20-Ga) is shorter than that found for 20-In (2.4806(8) Å).^42^

**Fig. 6:**
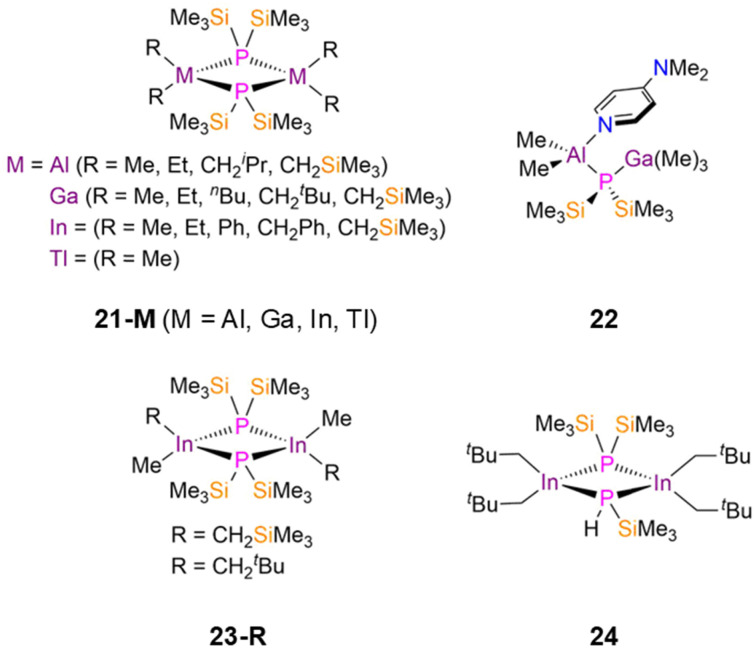
Dinuclear group 13 P′′ complexes [M(R)_2_(μ-P′′)]_2_ (21-MR; M = Al, Ga, In, Tl; R = Me, Et, Ph, ^*n*^Bu, CH_2_Ph, CH_2_^i^Pr, CH_2_^*t*^Bu, CH_2_SiMe_3_), [Al(Me)_2_(dmap)(μ-P′′)Ga(Me)_3_] (22), [In(Me)(R)(μ-P′′)]_2_ (23-R; R = CH_2_SiMe_3_, CH_2_^*t*^Bu), [{In(CH_2_^*t*^Bu)_2_}_2_(μ-P′′){μ-PH(SiMe_3_)}] (24).

There have been numerous published examples of symmetric dimeric group 13 P′′ complexes, [M(R)_2_(μ-P′′)]_2_ (21-MR; M = Al, Ga, In, Tl; R = Me, Et, Ph, ^*n*^Bu, CH_2_Ph, CH_2_^i^Pr, CH_2_^*t*^Bu, CH_2_SiMe_3_, Fig. 6), as these have found interest as precursors for III–V semiconductor materials.^43–57^ Each of these complexes consists of two four-coordinate distorted tetrahedral metal centres bridged by two P′′ ligands to give M_2_P_2_ cores. In 21-AlR the mean Al–P bond lengths increase as the steric influence of the R group increases: 21-AlMe, 2.457(2) Å; 21-AlEt, 2.458(1) Å; 21-AlCH_2_^i^Pr, 2.476(2) Å; 21-AlCH_2_SiMe_3_, 2.483(1) Å.^43–46^ This trend is also seen for the 21-GaR series: 21-GaMe, 2.450(1) Å; 21-GaEt, 2.4558(7) Å; 21-Ga^*n*^Bu, 2.4533(6) Å; 21-GaCH_2_^*t*^Bu, 2.517(3) Å; 21-GaCH_2_SiMe_3_, 2.4887(16) Å.^40,47–51^ Finally, the In–P bond lengths in 21-InR vary similarly: 21-InMe, 2.630(1) Å; 21-InEt, 2.645(1) Å; 21-InPh, 2.612(1) Å; 21-InCH_2_Ph, 2.6123(6) Å; 21-CH_2_SiMe_3_, 2.655(3) Å.^52–55^ The variation of R groups in these complexes changes their functionalities; for example, [In(^*t*^Bu)_2_(μ-P′′)]_2_ (21-In^*t*^Bu) was employed in the fabrication of nanowires of approximately 10–100 nm diameter.^55^ In 2003 Schulz disclosed that the combination of 17-Al with GaMe_3_ in the presence of dmap at −30 °C yielded the adduct [Al(Me)_2_(dmap)(μ-P′′)Ga(Me)_3_] (22, Fig. 6), as well as [Ga(Me)_2_(μ-P′′)]_2_ (21-GaMe) [AlMe_3_(dmap)]; the latter products formed upon decomposition when warming to room temperature as a result of a methyl group transfer from Ga to Al with concomitant Al–P bond cleavage.^58^ This rearrangement was monitored by VT ^31^P{^1^H} NMR spectroscopy; from −50 to −10 °C there was only a single resonance corresponding to 22 (−262 ppm), but above −10 °C an additional signal appears at −219 ppm due to the formation of 21-GaMe.^58^

In both 1992 and 1993, Wells reported the synthesis of three dimeric In(iii) P′′ complexes, [In(Me)(R)(μ-P′′)]_2_ (23-R; R = CH_2_SiMe_3_, CH_2_^*t*^Bu, Fig. 6) and [{In(CH_2_^*t*^Bu)_2_}_2_(μ-P′′){μ-PH(SiMe_3_)}] (24, Fig. 6).^59,60^ Complexes 23-R exhibit puckered In_2_P_2_ cores, with each In atom bound to two different alkyl groups, and mean In–P bond lengths of 2.635(2) Å (23-CH_2_SiMe_3_) and 2.637(3) Å (23-CH_2_^*t*^Bu).^59,60^ Complex 24 was isolated as a minor product from the reaction of [In(CH_2_^*t*^Bu)_2_(Cl)] and P(SiMe_3_)_3_, and represented the first example of a dinuclear In phosphide complex in which the phosphorus centres were substituted asymmetrically; complex 24 exhibits a mean In–P bond length of 2.650(3) Å and hydrocarbon solutions of this complex were found to be unstable at room temperature.^60^

**Fig. 7:**
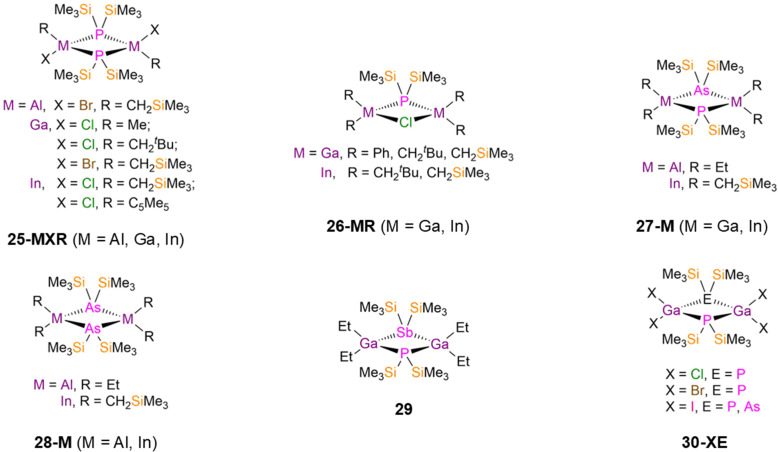
Dimeric group 13 P′′ complexes [M(R)(X)(μ-P′′)]_2_ (25-MXR: M = Al, Ga, In; X = Cl, Br; R = Me, CH_2_^*t*^Bu, CH_2_SiMe_3_, η^1^-Cp*; Cp* = C_5_Me_5_), [{M(R)_2_}_2_(μ-P′′)(μ-Cl)] (26-MR: M = Ga, In; R = Ph, CH_2_^*t*^Bu, CH_2_SiMe_3_), [{M(R)_2_}_2_(μ-P′′)(μ-As′′)] (27-M: M = Al, R = Et, M = In, R = CH_2_SiMe_3_), [M(R)_2_(μ-As′′)]_2_ (28-M: M = Al, R = Et, M = In, R = CH_2_SiMe_3_), [{Ga(Et)_2_}_2_(μ-P′′)(μ-Sb′′)] (29), [{Ga(X)_2_}_2_(μ-P′′){μ-E(SiMe_3_)_2_}] (30-ClP: X = Cl, Pn′′ = P′′; 30-BrP: X = Br, Pn′′ = P′′; 30-IP: X = I, Pn′′ = P′′; 30-IAs: X = I, Pn′′ = As′′).

There are numerous examples of dinuclear group 13 bridged P′′ complexes that contain either terminal or bridging halides as these are often used as precursors to functionalised complexes *via* salt metathesis reactions. For example, [M(R)(X)(μ-P′′)]_2_ (25-MXR; M = Al, Ga, In; X = Cl, Br; R = Me, CH_2_^*t*^Bu, CH_2_SiMe_3_, η^1^-Cp*; Cp* = C_5_Me_5_, Fig. 7) were synthesised from parent [M(R)(X)_2_] with either P(SiMe_3_)_3_ or LiP′′ between 1991–2004 by Schulz, Wells, and Theopold.^50,59,61–63,126^ Each of these complexes has a M_2_P_2_ core, with the four-coordinate group 13 metals having distorted tetrahedral geometries.^50,59,61–63,126^ Complex 25-AlBrCH_2_SiMe_3_ exhibits a mean Al–P bond length of 2.436(4) Å, whilst the Ga–P bond lengths of 25-GaXR range from 2.4106(10)–2.424(3) Å, and the In–P distances of 25-InXR vary from 2.593(2)–2.621(2) Å.^50,59,61,63^ Wells reported several examples of group 13 metal P′′ complexes where a chloride has been incorporated into the central four membered ring, [{M(R)_2_}_2_(μ-P′′)(μ-Cl)] (26-MR; M = Ga, In; R = Ph, CH_2_^*t*^Bu, CH_2_SiMe_3_, Fig. 7).^50,56,60,64^ For 26-GaCH_2_^*t*^Bu, the central Ga_2_PX core is planar, but 26-GaCH_2_SiMe_3_ deviates from planarity by 23° and 26-GaCH_2_Ph deviates by 6.4° due to steric effects.^50,64^ In contrast, the In P′′ complexes 26-InCH_2_^*t*^Bu and 26-InCH_2_SiMe_3_ were found to be planar, due to the increased size of In *vs*. Ga allowing for the large steric bulk of both alkyl and silylphosphide ligands.^56,60^ This increase in size from Ga to In is reflected in the respective mean M–P bond lengths; 26-GaR (2.391(4)–2.451(3) Å) and 26-InR (2.603(4)–2.622(5) Å).^50,56,60,64^

There are also examples of dinuclear group 13 P′′ complexes that contain bridging {Pn(SiMe_3_)_2_} ligands; Pn = As (As′′) or Sb (Sb′′). In 1994 and 1995, Wells synthesised the respective Al and In P′′ complexes [{M(R)_2_}_2_(μ-P′′)(μ-As′′)] (27-Al: M = Al, R = Et; 27-In: M = In, R = CH_2_SiMe_3_; Fig. 7) by the separate reactions of [M(R)_2_(P′′)]_2_ with [M(R)_2_(As′′)]_2_ (28-Al: M = Al, R = Et; 28-In: M = In, R = CH_2_SiMe_3_, Fig. 7),^65,66^ whilst an analogous Ga complex, [{Ga(Et)_2_}_2_(μ-P′′)(μ-Sb′′)] (29, Fig. 7), was synthesised by Wells in 2000 *via* the combination of [Ga(Et)_2_(Cl)] with P(SiMe_3_)_3_ and Sb(SiMe_3_)_3_; the As analogue of 29 was not structurally authenticated.^48^ Complexes 27-M and 29 represent rare examples of two different group 15 elements bridging between Al, Ga or In; in the solid state planar central M_2_PE cores (M = Al, In, E = As; M = Ga, E = Sb) were observed in each case, with the metal centres displaying distorted tetrahedral geometries.^48,65,66^ The mean M–P bond lengths increase with the size of the metal (27-Al: 2.497(1) Å; 29: 2.574(2) Å; 27-In: 2.691(3) Å).^48,65,66^ Between 1993–1995, Wells reported several other examples of dimeric Ga(iii) P′′ halide complexes, namely [{Ga(X)_2_}_2_(μ-P′′)(μ-Pn′′)] (30-ClP: X = Cl, Pn′′ = P′′; 30-BrP: X = Br, Pn′′ = P′′; 30-IP: X = I, Pn′′ = P′′; 30-IAs: X = I, Pn′′ = As′′; Fig. 7).^67–69^ These complexes were isolated from the separate reactions of GaX_3_ (X = Cl, Br, I) with either two equivalents of P(SiMe_3_)_3_ (30-ClP, 30-BrP, 30-IP) or one equivalent each of P(SiMe_3_)_3_ and As(SiMe_3_)_3_ (30-IAs).^67–69^ These complexes are the first examples of Ga pnictide dimers that contain only exocyclic halogen ligands, and in common with the previously discussed bridging pnictide complexes they all display a planar central Ga_2_PE core with the Ga atoms exhibiting distorted tetrahedral geometries. The mean Ga–P bond lengths range from 2.379(3)–2.398(4) Å, with the increase in size of the terminal halogen leading to longer bridging Ga–P bond distances.^67–69^ The incorporation of the bridging As′′ ligand in 30-IAs gives a longer Ga–P bond length (2.443(3) Å) than the mean Ga–P distance in 30-IP (2.398(4) Å).^69^ The authors anticipated that these complexes would be viable starting materials for introducing further substitution at the Ga centres through dehalosilylation and salt elimination reactions.^67–69^

**Fig. 8:**
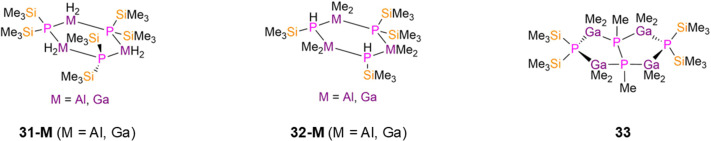
Polynuclear 13 P′′ complexes [M(H)_2_(μ-P′′)]_3_ (31-M; M = Al, Ga), [{M(Me)_2_}_3_(μ-P′′){HP(SiMe_3_)}_2_] (32-M; M = Al, Ga), [{Ga(Me)_2_(μ-P′′)}_2_{μ-P(Me)}]_2_ (33).

Although there are numerous reports of dimeric group 13 P′′ complexes, there are just a handful of structurally characterised tri- or tetra-nuclear examples. The trinuclear complexes [M(H)_2_(μ-P′′)]_3_ (M = Al, 31-Al; M = Ga, 31-Ga, Fig. 8), were reported by Wells in 1997 and 1998, and [{M(Me)_2_}_3_(μ-P′′){HP(SiMe_3_)}_2_] (M = Al, 32-Al; M = Ga, 32-Ga, Fig. 8), were reported by Robinson and Weidlein in 1994 and 1999, respectively.^70–72^ These complexes were synthesised by the reaction of parent H_3_M·NMe_3_ (M = Al, Ga) with P(SiMe_3_)_3_ for 31-M, or from parent [M(Me)_2_(OMe)]_3_ (M = Al, Ga) and HP′′ for 32-M. Each complex features six-membered M_3_P_3_ rings with three four-coordinate distorted tetrahedral Al/Ga atoms, with 31-M exhibiting planar rings and 32-M having twisted conformations in order to relieve steric congestion.^70–72^ The mean M–P bond lengths increase from 31-M to 32-M: 2.398(4) Å (31-Al), 2.4287(13) Å (32-Al), 2.392(3) Å (31-Ga), and 2.415(2) Å (32-Ga).^70–72^ The tetranuclear Ga(iii) P′′ complex [{Ga(Me)_2_(μ-P′′)}_2_{μ-P(Me)}]_2_ (33, Fig. 8), reported by Robinson in 1994, was prepared from the reaction of the Lewis acid–base adduct [GaMe_3_-PMe_3_] with P(SiMe_3_)_3_.^73^ Complex 33 is bicyclic and features two fused Ga_2_P_3_ five-membered rings, with distorted tetrahedral geometries of both Ga and P atoms, mean Ga–P bond lengths of 2.443(9) Å and a P–P single bond distance of 2.25(3) Å.^73^ Remarkably, unlike the vast majority of P′′ complexes, 31-M, 32-M and 33 are all stable in air for a limited amount of time.^70–73^

##### 2.2.1.1 Heterometallic group 13 complexes

**Fig. 9:**
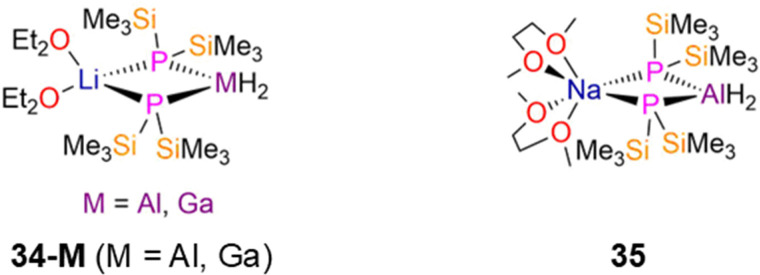
Heterometallic group 13 P′′ complexes [M(H)_2_(μ-P′′)_2_Li(Et_2_O)_2_] (34-M; M = Al, Ga), [Al(H)_2_(μ-P′′)_2_Na(DME)_2_] (35).

There are a handful of structurally characterised examples of heterometallic mixed s- and p-block P′′ complexes, which are often used as precursors for complexes that possess bonds between group 13–15 elements.^74^ The Li group 13 ‘ate’ P′′ complexes, [M(H)_2_(μ-P′′)_2_Li(Et_2_O)_2_] (34-M; M = Al, Ga; Fig. 9) were synthesised by Wells in 1997 and 1998, respectively, *via* the reaction of LiMH_4_ with two equivalents of P(SiMe_3_)_3_ (M = Al, Ga) in diethyl ether.^74,75^ The LiP_2_M (M = Al, Ga) cores of these complexes were confirmed to be planar by both NMR spectroscopy and single crystal XRD, and show mean M–P bond lengths of 2.4001(13) Å (34-Al) and 2.4122(12) Å (34-Ga).^74,75^ Both the hydrogen and carbon atoms of the SiMe_3_ groups gave triplet resonances in the ^1^H and ^13^C{^1^H} NMR spectra from second order coupling to two identical ^31^P nuclei, and signals at −282.0 and −277.7 ppm were seen in the ^31^P{^1^H} NMR spectra of 34-Al and 34-Ga, respectively.^74,75^ The M–H bonds were evidenced by stretching absorptions in IR spectra at 1752 cm^−1^ (34-Al) and 1838 cm^−1^ (34-Ga), together with broad resonances in ^1^H NMR spectra at 4.3 ppm (34-Al) and 4.7 ppm (34-Ga).^74,75^ A structurally analogous Al P′′ complex, [Al(H)_2_(μ-P′′)_2_Na(DME)_2_] (35, Fig. 9), was synthesised by Hänisch in 2004 *via* the reaction of NaAlH_4_ with four equivalents of the primary phosphine, H_2_PSiMe_3_ in DME.^76^ Similarly to 34-M, complex 35 has a planar four-membered NaP_2_Al ring, mean Al–P bond lengths of 2.405(1) Å, triplet resonances in both its ^1^H and ^13^C{^1^H} NMR spectra for the SiMe_3_ groups, and a single resonance at −283.0 ppm in its ^31^P{^1^H} NMR spectrum. Both symmetric and asymmetric stretches of the Al–H bonds were observed in the IR spectrum of 35 at 1762 and 1728 cm^−1^.^76^

#### 2.2.2 Homometallic group 14 complexes

**Fig. 10:**
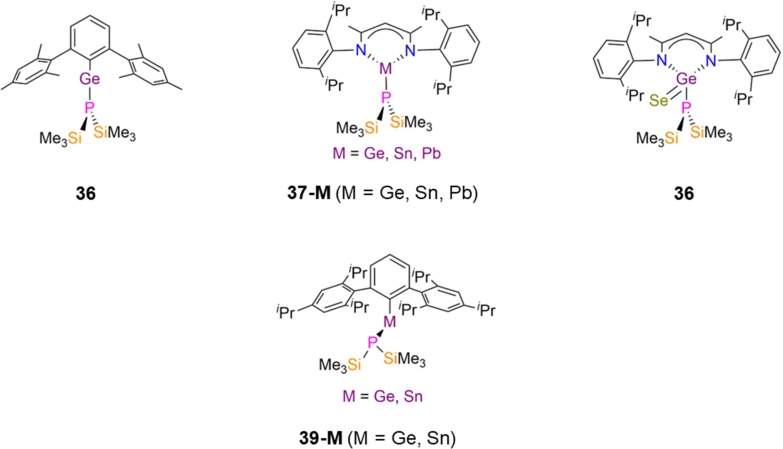
Mononuclear group 14 P′′ complexes [Ge(P′′)(Ar^Mes^)] (Ar^Mes^ = C_6_H_3_Mes_2_-2,6) (36), [M(^Dipp^Nacnac)(P′′)] (37-M; M = Ge, Sn, Pb, ^Dipp^Nacnac = CH{(CH_3_)_2_CN(Dipp)}_2_; Dipp = C_6_H_3_^i^Pr_2_-2,6), [Ge(Se)(^Dipp^Nacnac)(P′′)] (38), [M(Ph*)(P′′)] (39-M; M = Ge, Sn, Ph* = C_6_H_3_Trip-2,6, Trip = C_6_H_2_^i^Pr_3_-2,4,6).

Group 14 P′′ complexes are less common than group 13 examples, but have received particular interest as σ-donor ligands, and as frustrated Lewis pairs, due to their ability to mediate E–H bond activation.^127–133^ The terphenyl Ge(ii) P′′ complex [Ge(P′′)(Ar^Mes^)], Ar^Mes^ = C_6_H_3_Mes_2_-2,6 (36, Fig. 10), synthesised by Aldridge in 2016, features a two-coordinate Ge atom with a pyramidal phosphorus centre displaying a C_Ar_–Ge–P angle of 106.1(1)° and a Ge–P bond length of 2.329(1) Å.^77^ It was noted that this complex was unreactive towards H_2_ and NH_3_, and as such is not well-suited for promoting small molecule activation reactions.^77^ Complexes of the type [M(^Dipp^Nacnac)(P′′)] (M = Ge, 37-Ge; Sn, 37-Sn; Pb, 37-Pb; Fig. 10) have also been explored by several groups as σ-donor ligands.^78–80^ These complexes exist as both *endo*- and *exo*-conformers in the solid state, with the *endo*-conformers exhibiting planar phosphorus centres (37-Ge and 37-Sn) and the *exo*-conformers possessing pyramidal phosphorus centres.^78–80^ Complexes 37-M exhibit increasing M–P bond lengths with the size of the metal centre: 37-Ge, 2.3912(8) Å; 37-Sn, 2.5526(7) Å; 37-Pb, 2.715(2) Å.^78–80^ The ^31^P NMR spectra of the 37-M triad respectively showed signals at −192.7, −183.5 (^1^*J*_SnP_ = 2427 Hz; assume mean coupling constants from 7.7% abundant ^117^Sn and 8.6% abundant ^119^Sn coupling constants, both *I* = ½) and −116.6 (^1^*J*_PbP_ = 1580 Hz, 22.1% abundant ^207^Pb *I* = ½) ppm, with the Sn centres of 37-Sn being planar and the Pb centres of 37-Pb being pyramidal.^78–80^ In 2017 Fulton reported that the addition of elemental Se to 37-Ge gave the Ge(iv) selenide complex, [Ge(Se)(^Dipp^Nacnac)(P′′)], (38, Fig. 10), which displayed a Ge–P bond length (2.2976(7) Å) that is shorter than that of 37-Ge.^78,79,81^ A single resonance was observed in the ^31^P{^1^H} NMR spectrum of 38 at −176.2 ppm, with both selenium and silicon satellites (^1^*J*_SeP_ = 52 Hz, 7.6% abundant ^77^Se *I* = ½, and ^1^*J*_PSi_ = 26 Hz).^81^ In 2010, Scheer reported the terphenyl Ge(ii) and Sn(ii) P′′ complexes, [M(Ph*)(P′′)] (M = Ge, 39-Ge; Sn, 39-Sn; Ph* = C_6_H_3_Trip-2,6, Trip = C_6_H_2_^i^Pr_3_-2,4,6; Fig. 10), which were synthesised by a redox reaction of [M(Ph*)(X)_3_] (M = Ge, X = Cl; M = Sn, X = Cl, Br) with LiP′′.^82^ Both complexes display a *trans*-bent geometry, as commonly seen for germylene/stannylene complexes, with the phosphorus atom exhibiting a distorted trigonal pyramidal geometry and bond lengths of 2.291(4) Å (39-Ge) and 2.527(1) Å (39-Sn), signifying that the P lone pair is not donating to the group 14 centre.^82^

**Fig. 11:**
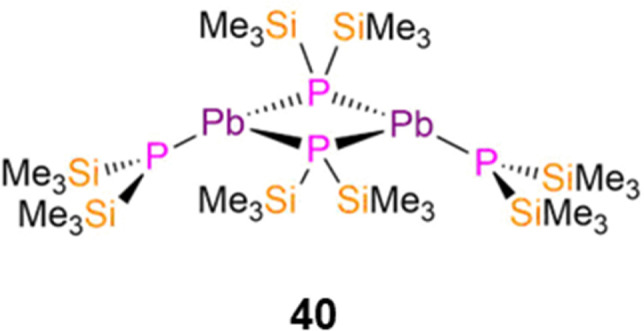
Complex [Pb(P′′)(μ-P′′)]_2_ (40).

In 1993, Buhro reported the dimeric Pb(ii) P′′ complex, [Pb(P′′)(μ-P′′)]_2_ (40, Fig. 11) which was synthesised by the protonolysis reaction of [Pb(N′′)_2_] with two equivalents of HP′′; the Sn(ii) analogue of 40 was synthesised by the same methods but was not structurally authenticated.^83^ Complex 40 was reported to decompose to Pb and the diphosphine (Me_3_Si)_2_P–P(SiMe_3_)_2_ in refluxing benzene. Complex 40 exhibits a puckered central M_2_P_2_ core and crystallises with the trigonal pyramidal terminal P′′ ligands mutually *syn*-, with mean Pb–P bond lengths of 2.77(1) Å (terminal) and 2.70(1) Å (bridging).^83^ An equilibrium between the *syn*- and *anti*-conformers of 40 is seen in solution, with the ^31^P{^1^H} NMR spectrum containing two sets of resonances in a 2 : 1 and 1 : 1 ratio (−217.3 ppm, ^1^*J*_PbP_ = 1264 Hz, *anti*-terminal; −218.0 ppm, ^1^*J*_PbP_ = 1183 Hz, *syn*-terminal; −281.4 ppm, ^1^*J*_PbP_ = 1598 Hz, *syn*-bridging; −302.4 ppm, ^1^*J*_PbP_ = 1658 Hz, *anti*-bridging).^83^ Further assignment of these conformers was enabled by ^1^H NMR spectroscopy as the SiMe_3_ groups on the bridging P′′ are inequivalent for the *syn*- and equivalent for the *anti*-conformer, thus three resonances are associated with the former and two for the latter conformer.^83^ It was deduced from the intensities of the resonances at room temperature that solutions of 40 contained nearly equal amounts of *syn*- and *anti*-conformers.^83^

##### 2.2.2.1 Heterometallic group 14 complexes

**Fig. 12:**
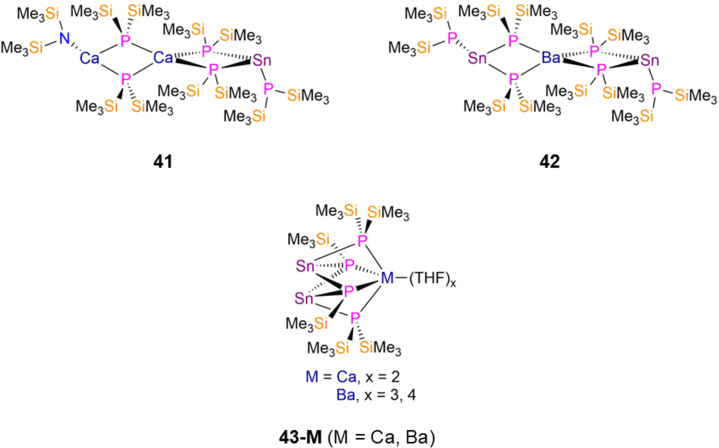
Heterometallic group 14 P′′ complexes [Sn(P′′)(μ-P′′)_2_Ca(μ-P′′)_2_Ca(N′′)] (41), [{Sn(P′′)(μ-P′′)_2_}_2_Ba] (42), [M(THF)_*x*_{Sn(μ_2_-P′′)(μ_3_-P′)}_2_] (43-M; M = Ca, *x* = 2, M = Ba, *x* = 3, 4, P′ = {P(SiMe_3_)}).

In 1994 Westerhausen reported the synthesis of two Sn(ii) P′′ complexes, [Sn(P′′)(μ-P′′)_2_Ca(μ-P′′)_2_Ca(N′′)] (41, Fig. 12) and [{Sn(P′′)(μ_2_-P′′)_2_}_2_Ba] (42, Fig. 12), by the separate reactions of the respective M(ii) group 2 bis-amide complexes, [M(N′′)_2_] (M = Ca, Ba) with [Sn(N′′)_2_] and five or six equivalents of HP′′ in toluene.^34,84^ Both complexes exhibit central {M(μ-P′′)_4_} (M = Ca, Ba) cores, with 41 featuring one terminal CaN′′ and one terminal SnP′′ fragment, and 42 having two terminal SnP′′ moieties.^34,84^ The central s-block atom of both complexes have distorted tetrahedral geometries, the terminal Ca atom in 41 is planar, and the terminal Sn atoms of both 41 and 42 are pyramidal. The bridging Sn–P bond distances in 41 are longer than that of the terminal Sn–P bond by approximately 0.09 Å, whereas the corresponding difference in 42 is *ca*. 0.11 Å.^34,84^ The cluster complexes, [M(THF)_*x*_{Sn(μ_2_-P′′)(μ_3_-P′)}_2_] (M = Ca, *x* = 2, 43-Ca; M = Ba, *x* = 3, 4, 43-Ba, P′ = {P(SiMe_3_)}; Fig. 12), were synthesized by Westerhausen in 1994 and 1995 using analogous methods but with THF as the reaction solvent. Both 43-M feature two three-coordinate Sn atoms, each bound to one bridging P′′ and two dianionic bridging silylphosphadiides, {P(SiMe_3_)}, to the respective s-block metal; 43-Ca and 43-Ba have mean Sn–P bond lengths of 2.642(1) Å and 2.885(9) Å, respectively.^33,34^

### 2.3 d-Block P′′ complexes

d-Transition metal P′′ complexes are known to be versatile reagents for the formation of alkylidene-phosphides,^134,135^ diphosphenyls^136^ and cyclophosphines;^137^ bulky supporting ligands are required in order to prevent oligomerisation to give monomeric complexes, and for homoleptic complexes oligomers tend to form. As expected and in common with the vast majority of other ligands,^22,23^ first row d-transition metal P′′ chemistry is more developed than for the heavier second and third row metals. It is noteworthy that no group 5 or 9 P′′ complexes were found in the CSD search described above, and there are also no structurally authenticated Tc, Re, Ru or Os P′′ complexes,^9^ thus there are multiple opportunities here to expand d-block P′′ chemistry rapidly.

#### 2.3.1 Group 3 complexes

**Fig. 13:**
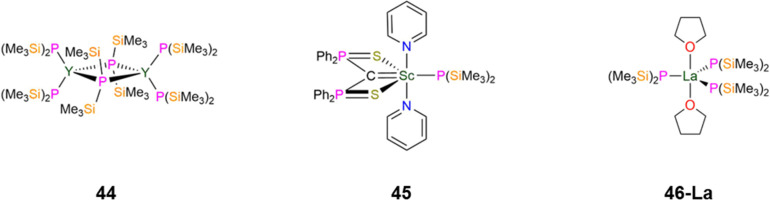
Group 3 P′′ complexes [{Y(P′′)_2_}_2_(μ-P′′)_2_] (44), [Sc{C(PPh_2_S)_2_}(P′′)(py)_2_] (45), [La{P(SiMe_3_)_2_}_3_(THF)_2_] (46-La).

In 1998, Westerhausen synthesised the dimeric Y complex, [{Y(P′′)_2_}_2_(μ-P′′)_2_] (44, Fig. 13) with the crystal structure being reported in 2002, by the protonolysis reaction of [Y{CH(SiMe_3_)_2_}_3_] with three equivalents of HP′′.^86,87^ Both Y(iii) cations in 44 are bound by two terminal and two bridging P′′ in distorted tetrahedral geometries; the mean Y–P_terminal_ bond lengths (2.678(4) Å) are shorter than the mean Y–P_bridging_ bond lengths (2.849(4) Å) as expected.^86,87^ These metrical parameters may be compared to those of a structurally analogous dimeric Y(iii) amide complex, [Y{N(SiHMe_2_)_2_}_2_{μ-N(SiHMe_2_)_2_}]_2_, which has mean Y–N_terminal_ bond lengths of 2.249(4) Å and mean Y–N_bridging_ bond lengths of 2.479(4) Å.^138^ The dimeric structure of 44 was shown to be maintained in solution by ^31^P{^1^H} NMR spectroscopy, with a doublet of pentets observed at −107.8 ppm (^1^*J*_YP_ = 56.7 Hz, ^2^*J*_PP_ = 5.0 Hz) for bridging P′′ and a doublet of triplets −104.8 ppm (^1^*J*_YP_ = 122.4 Hz, ^2^*J*_PP_ = 5.0 Hz) for terminal P′′.^86,87^

In 2015, as part of a study by Mézailles into how the anionic ligand *trans*- to a bound carbon atom in Sc(iii) methanediide complexes influences complex geometry, the P′′ complex, [Sc{C(PPh_2_S)_2_}(P′′)(py)_2_] (45, Fig. 13) was synthesised.^88^ Complex 45 has a Sc–P bond length of 2.618(14) Å; it was found that the methanediide carbon atom is planar for all Sc(iii) complexes explored in this study save the N′′ analogue, [Sc{C(PPh_2_S)_2_}(N′′)(THF)], which has one THF bound to Sc rather than two pyridines. Density Functional Theory (DFT) calculations indicated that the coordinating solvent made little impact on the planarity of the methanediide. Natural Bond Orbital (NBO) analysis revealed that in 45 the donation from both the C and P atoms are stronger than the corresponding C and N atoms in [Sc{C(PPh_2_S)_2_}(N′′)(THF)], which leads to the difference in methanediide geometry.^88^ In 2024, Mills and co-workers reported the synthesis of [La{P(SiMe_3_)_2_}_3_(THF)_2_] (46-La, Fig. 13) by a salt metathesis reaction of the respective [La(i)_3_(THF)_4_] starting material with three equivalents of KP′′.^89^ Single crystal XRD showed that 46-La to exhibit a distorted trigonal bipyramidal geometry, with three equatorial P′′ and two axial THF molecules, with the O–La–O angle showing a small deviation from linearity: 175.14(8)° and a mean La–P bond length of 2.886(2) Å. Two of the equatorial P′′ in 46-La show pyramidal geometries about the phosphorus atom, whereas the third P′′ exhibits a planar geometry. Solid state ^31^P MAS NMR spectroscopy of 46-La revealed two components in their spectra in a 2 : 1 ratio, with the major component assigned to pyramidal and the minor component to planar P environments. This is in contrast to the solution ^31^P{^1^H} NMR spectrum of 46-La, which exhibits one broad resonance due to dynamic processes and quadrupolar broadening due to the 99.9% abundant ^77^La *I* = 7/2 nuclei.

#### 2.3.2 Group 4 complexes

**Fig. 14:**
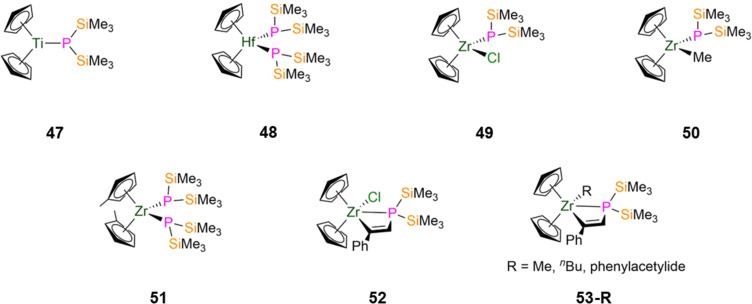
Group 4 P′′ complexes [Ti(Cp)_2_(P′′)] (47), [Hf(Cp)_2_(P′′)_2_] (48), [Zr(Cp)_2_(Cl)(P′′)] (49), [Zr(Cp)_2_(Me)(P′′)] (50), [Zr(C_5_H_4_Me)_2_(P′′)_2_] (51), [Zr(Cp)_2_(Cl){P(SiMe_3_)_2_C(H)C(Ph)}] (52), [Zr(Cp)_2_(R){P(SiMe_3_)_2_C(H)C(Ph)}] (53-R; R = Me, ^*n*^Bu, C≡CPh).

The first d-block P′′ complex to the best of our knowledge, [Ti(NMe_2_)_3_(P′′)], was reported in 1970 by Bürger, but this was not structurally authenticated.^18^ In 1991, the paramagnetic bis-Cp Ti(iii) P′′ complex, [Ti(Cp)_2_(P′′)] (47, Fig. 14), was synthesised by Fenske by the salt metathesis reaction of [Ti(Cp)(Cl)] with LiP′′.^90^ This complex features a planar phosphorus atom as a result of the π-interaction between Ti and P, and has a short Ti–P bond length of 2.468(1) Å, indicating some double bond character, although the sum of covalent radii of a Ti–P single bond (2.47 Å) matches that reported for 47.^139^ The ^31^P{^1^H} NMR spectrum of 47 exhibits a single broad resonance at 251 ppm, and an isotropic signal was observed in the EPR spectrum with a *g*-value of 1.9779.^90^ In 1987 Weber reported a series of heavy group 4 M(iv) P′′ complexes with two ancillary Cp ligands by salt metathesis methodologies.^91^ The reaction of [Zr(Cp)_2_(Cl)_2_] with one or two equivalents of LiP′′ exclusively gave [Zr(Cp)_2_(P′′)_2_] (48-Zr), whilst for the analogous reactions of the Hf analogue [Hf(Cp)_2_(Cl)_2_] with LiP′′ rapid substitution of the first chloride gave [Hf(Cp)_2_(Cl)(P′′)] (49-Hf), and the second substitution proceeds more slowly to give [Hf(Cp)_2_(P′′)_2_] (48-Hf, Fig. 14). Complexes 48-Zr and 49-Hf were not structurally characterised in this study, but in 1988 Lappert reported the synthesis of [Zr(Cp)_2_(Cl)(P′′)] (49-Zr, Fig. 14) from the reaction of [Zr(Cp)_2_(Cl)_2_] with [Li(μ-P′′)(THF)_2_]_2_.^91,92^ The reaction of [Zr(Cp)_2_(Me)(Cl)] with LiP′′ gave the monophosphide complex, [Zr(Cp)_2_(Me)(P′′)] (50, Fig. 14).^91^ All of these structurally characterised group 4 M(iv) P′′ complexes exhibit *pseudo*-tetrahedral geometries and additionally show some interesting structural features. For example, in 48-Hf it was seen that one P′′ has a planar geometry with a Hf–P bond length of 2.553(1) Å, whilst the other P′′ is pyramidal with a longer Hf–P distance of 2.654(1) Å.^91^ The shorter Hf–P bond length in the planar P′′ arises from its orthogonal lone pair being involved in π-bonding with Hf.^91^ It was also noted in the ^31^P{^1^H} NMR spectrum of 48-Hf that this structural feature is maintained in solution, with two resonances seen at −69.90 and −98.83 ppm for the pyramidal and planar P′′, respectively.^91^ For complex 49-Zr, a relatively short Zr–P bond length (2.547(6) Å) is seen for the near-planar P′′ (Σ angles = 344.4°), though this is slightly pyramidalized compared to the planar P′′ in 48-Hf (Σ angles = 360°).^92^ In 1992, Hey-Hawkins synthesised a derivative of 48-Zr, [Zr(C_5_H_4_Me)_2_(P′′)_2_] (50, Fig. 14), by the salt metathesis reaction of [Zr(C_5_H_4_Me)_2_(Cl)_2_] with LiP′′.^93^ Complex 50 shows a pseudo-tetrahedral geometry, where both phosphorus atoms are planar and the Zr–P bond lengths are longer than the corresponding distances in 49-Zr (2.600(2) and 2.634(2) Å).^93^ VT ^31^P{^1^H} NMR spectra of 51 indicated that the planarity at phosphorus was retained in solution, with two equivalent P atoms evidenced by a single resonance at −75.3 ppm at 298 K, which shifts to −86.2 ppm at 173 K.^93^

Between 1992–1995, Hey-Hawkins synthesised zirconocene alkenyl complexes that feature P′′ incorporated into metallacycles.^94,95^ The insertion reaction of [Zr(Cp)_2_(P′′)(Cl)] with phenylacetylene in toluene at reflux gave [Zr(Cp)_2_(Cl){P(SiMe_3_)_2_C(H)C(Ph)}] (52, Fig. 14), which can undergo subsequent salt metathesis reactions with RLi (R = Me, ^*n*^Bu, C≡CPh) to yield [Zr(Cp)_2_(R){P(SiMe_3_)_2_C(H)C(Ph)}] (R = Me, ^*n*^Bu, C≡CPh, 53-R; Fig. 14); 53-^*n*^Bu was not structurally characterised.^94,95^^31^P{^1^H} NMR spectroscopy showed modest changes in chemical shifts when Cl is substituted for an R group (R = Cl, −179.5 ppm; Me, −179.3 ppm; ^*n*^Bu, −176.6 ppm; C≡CPh, −189.9 ppm).^94,95^ For all of these complexes a doublet is observed in the ^1^H NMR spectra for the alkenyl proton, with ^1^*J*_PH_ coupling constants ranging from 8–12 Hz.^94,95^ The alkenyl moieties exhibit the *Z*-configuration in the solid state, and the Zr–P distances (52: 2.855(4) Å; 53-Me: 2.915(3) Å; 53-C≡CPh: 2.774(3) Å) indicate that Zr–P interactions persist in solution for these complexes.^94,95^ The phosphorus atoms exhibit pyramidal geometries in 52 and 53-R, due to steric effects causing wide Si–P–Si angles.^94,95^

#### 2.3.3 Group 6 complexes

**Fig. 15:**
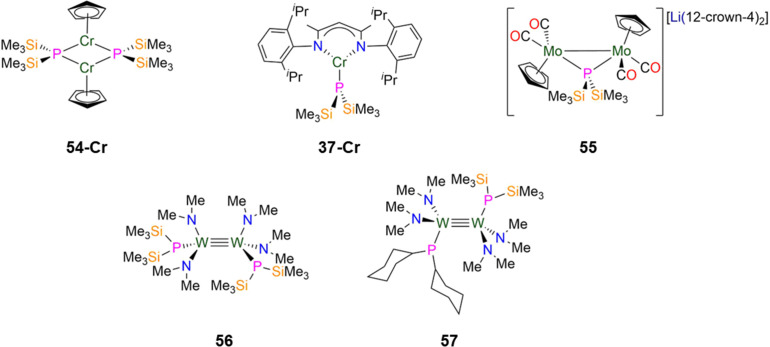
Group 6 P′′ complexes [Cr(Cp)(μ-P′′)]_2_ (54-Cr), [Cr(^Dipp^Nacnac)(P′′)] (37-Cr; ^Dipp^Nacnac = CH{(CH_3_)_2_CN(Dipp)}_2_; Dipp = C_6_H_3_^i^Pr_2_-2,6), [Li(12-crown-4)][{Mo(Cp)(CO)_2_}_2_(μ-P′′)] (55), [1,2-W_2_(P′′)(NMe_2_)_4_] (56), [1,2-W_2_(PCy_2_)(P′′)(NMe_2_)_4_] (57).

Dimeric mid-transition metal complexes have been investigated for their applications as molecular switches and molecular magnets due to the spin crossover and exchange coupling properties that these metals possess.^83,85,96^ In 2013, Layfield and Scheer reported the synthesis of the dimeric Cr(ii) Cp P′′ complex [Cr(Cp)(μ-P′′)]_2_ (54-Cr, Fig. 15) by the salt metathesis reaction of LiP′′ with [Cr(Cp)_2_].^96^ In the solid state each Cr atom of 54-Cr exhibits a pseudo-trigonal geometry, being bound to two phosphorus atoms and one η^5^-Cp ligand; the Cr–P bond lengths of 2.3814(9) and 2.3864(9) Å fall within the known range for cyclic Cr phosphide complexes.^9,85,96^ From magnetic susceptibility measurements, the high-temperature limit (*χ*_M_*T*) of 54-Cr was found to be 0.60 cm^3^ K mol^−1^, which is lower than the spin-only value of 6.0 cm^3^ K mol^−1^ for two non-interacting high-spin Cr(ii) ions and is indicative of strong antiferromagnetic coupling.^96^ This is also observed in variable temperature susceptibility measurements, where there is a steady decrease of *χ*_M_*T* towards zero upon cooling to 2 K; for the diamagnetic spin ground state where *S* = 0 the exchange coupling constant was calculated to be *J* = −166 cm^−1^.^96^

In 2024, Weigend and Hänisch synthesised a Cr(ii) P′′ complex, [Cr(^Dipp^Nacnac)(P′′)] (37-Cr, Fig. 15) by the salt metathesis reaction of the Cr(ii) precursor [Cr(^Dipp^Nacnac)(Cl)] with one equivalent of LiP′′ in Et_2_O at −78 °C.^140^ Complex 37-Cr features a nearly planar P atom (Σ angles = 356°), which allows its lone electron pair to act as a π-donor into a vacant Cr 3d orbital, leading to a short Cr–P bond length of 2.3641(3) Å.^140^

In 2021, Scheer synthesised the dinuclear Mo(i) P′′ complex, [Li(12-crown-4)_2_][{Mo(Cp)(CO)_2_}_2_(μ-P′′)] (55, Fig. 15) by the addition of LiP′′ to [Mo(Cp)(CO)_2_]_2_ in THF; the addition of one equivalent of either AsCl_3_ or SbCl_3_ to 55 gives the mixed-dipnictogen complexes [{Mo(Cp)(CO)_2_}_2_(μ-PE)] (E = As, Sb), releasing two equivalents of SiMe_3_Cl in the process.^97^ The Mo–P distances in 55 are 2.4304(6) Å, which is between that expected for a single or double bond, and the phosphorus atom exhibits a distorted tetrahedral geometry. A long Mo–Mo bond length in 55 (3.1890 Å) is observed compared to the starting material [Mo(Cp)(CO)_2_]_2_ (2.4477(12) Å), due to the degradation of the Mo≡Mo triple bond to a Mo–Mo single bond.^97^

In 1992, Chisholm synthesised two examples of dinuclear W(iii) complexes that exhibit tungsten-tungsten triple bonds and incorporate both P′′ and amide ligands.^98^ The salt metathesis reaction of [1,2-W_2_(Cl)_2_(NMe_2_)_4_] with two equivalents of LiP′′ gave [1,2-W_2_(P′′)_2_(NMe_2_)_4_] (56), whilst combination of the mono-substituted precursor [1,2-W_2_(Cl)(P′′)(NMe_2_)_4_] with LiPCy_2_ gave [1,2-W_2_(PCy_2_)(P′′)(NMe_2_)_4_] (57, Fig. 15).^98^ Complex 56 exhibits an *anti*-conformation in the solid-state across the triple bond, with trigonal planar nitrogen atoms and pyramidal phosphorus atoms.^98^ The W≡W triple bond lengths of 2.2989(9) Å (56) and 2.3016(10) Å (57) are similar to the mean values for W≡W triple bonds deposited on the CCDC (2.32(5) Å)^9^ and the W–P distances are statistically equivalent at 2.423(3) Å (56) and 2.425(4) Å (57).^98^ These complexes were characterised by both ^1^H and ^31^P{^1^H} NMR spectroscopy, with the latter showing a dynamic equilibrium in solution between the *anti*- and *gauche*-conformations; the ^31^P chemical shifts are −106.7 ppm (56) and −106.9 ppm (57) for the former and −88.3 ppm (56) and −96.3 ppm (57) for the latter.^98^ It was noted that upon lowering the temperature from 295 to 193 K, the ^31^P chemical shifts move to a higher field and a change of line shape is observed, which was attributed to inversion of the phosphorus centres.^98^

#### 2.3.4 Group 7 complexes

**Fig. 16:**
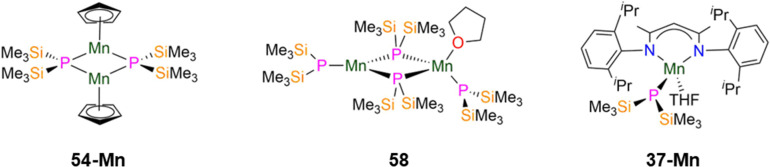
Group 7 P′′ complexes [Mn(Cp)(μ-P′′)]_2_ (54-Mn), [{Mn(P′′)(μ-P′′)_2_}{Mn(P′′)(THF)}] (58), [Mn(^Dipp^Nacnac)(P′′)(THF)] (37-Mn; ^Dipp^Nacnac = CH{(CH_3_)_2_CN(Dipp)}_2_; Dipp = C_6_H_3_^i^Pr_2_-2,6).

In 2012 Layfield and Scheer reported the synthesis of the Mn(ii) P′′ complex [[Mn(Cp)(μ-P′′)]_2_] (54-Mn, Fig. 16) by the salt metathesis reaction of LiP′′ with [Mn(Cp)_2_].^85^ The solid state structure of 54-Mn is analogous to the Cr(ii) complex 54-Cr discussed in the previous section, with the Mn–P bond lengths of 54-Mn being longer at 2.5075(5) and 2.5123(5) Å.^85^ Variable temperature magnetic measurements of 54-Mn showed that *χ*_M_*T* decreases from 5.71 cm^3^ K mol^−1^ at 300 K to 0.027 cm^3^ K mol^−1^ at 2 K, with the former value being approximately 75% of the expected value (8.75 cm^3^ K mol^−1^ at 300 K) for two *S* = 5/2 Mn(ii) ions. The small value of *χ*_M_*T* at 2 K was rationalised as the dimer having a diamagnetic ground state, likely due to antiferromagnetic exchange.^85^

In 1993, Buhro and co-workers reported the synthesis of the dinuclear Mn(ii) P′′ complex [{Mn(P′′)(μ-P′′)_2_}{Mn(P′′)(THF)}] (58, Fig. 16) from the protonolysis reaction of [Mn(N′′)_2_(THF)] with two equivalents of HP′′ in hexane.^83^ In the solid state 58 consists of one trigonal planar three-coordinate Mn centre with two bridging and one terminal P′′, and one four-coordinate distorted tetrahedral Mn centre with two bridging and one terminal P′′, together with a coordinated THF. It was noted that the bound THF in 58 can be removed when this complex is exposed to a dynamic vacuum.^83^ The central Mn_2_P_2_ core in 58 is nearly planar and the phosphorus atoms of the terminal P′′ adopt pyramidal geometries (mean Σ angles 334.2(3)°).^83^ The bridging Mn–P distances range from 2.493(2)–2.565(2) Å, whereas the terminal Mn–P bond lengths are 2.417(3) Å for the three-coordinate Mn and 2.461(2) Å for the four-coordinate Mn centre.^83^ Due to the paramagnetic nature of complex 58, NMR spectroscopic data were intractable, but Evans method magnetic susceptibility measurements gave a *μ*_eff_ value of 3.33*μ*_B_ per Mn atom; this is lower than the spin-only value for a high-spin system with *S* = 5/2 (5.92*μ*_B_) and was ascribed to the Mn centres being antiferromagnetically coupled.^83^

In 2024 Weigend and Hänisch synthesised the Mn(ii) P′′ complex [Mn(^Dipp^Nacnac)(P′′)(THF)] (37-Mn, Fig. 16), in an analogous manner to 37-Cr discussed in the previous section.^140^ The structure of 37-Mn is similar to 37-Cr but THF is additionally bound to Mn in the solid state; the Mn–P bond length of 37-Mn (2.461(1) Å) is longer than the corresponding Cr–P distance seen in 37-Cr and is similar to the mean Mn–P bond lengths found in 54-M (2.5075(5) Å) and 58 (2.461(2) Å).^83,85^

#### 2.3.5 Group 8 complexes

Although the syntheses of a number of group 8 P′′ complexes have been reported, remarkably only one structurally authenticated Fe(ii) P′′ complex has been reported to date.^99^ The first group 8 P′′ complex to be reported, [Fe(Cp)(CO)_2_(P′′)], was prepared by Schäfer in 1980 by the salt metathesis reactions of [Fe(Cp)(CO)_2_(X)] (X = Cl, Br) with Li P′′.^99*a*^ A reactivity study was reported in the same publication. The separate reactions of [Fe(Cp)(CO)_2_(P′′)] with [Fe_2_(CO)_9_] and [Ni(CO)_4_] gave the P′′-bridged complexes [{Fe(Cp)(CO)_2_}(μ-P′′){Fe(CO)_4_}] and [{Fe(Cp)(CO)_2_}(μ-P′′){Ni(CO)_3_}], respectively. Photolysis of the former complex was proposed to give [{Fe(Cp)(CO)}(μ-P′′)(μ-CO){Fe(CO)_3_}], whilst the treatment of either complex with stoichiometric methanol gave respective PH_2_-bridged products by cleavage of P–Si bonds. Between 1985 and 1987 Weber reported the synthesis of the group 8 M(ii) P′′ complexes [M(Cp*)(CO)_2_(P′′)] (M = Fe, Ru, Os) and [Fe(Cp*)(CO)(PPh_3_)(P′′)] by salt metathesis reactions;^99*b*–*e*^ reactions of these complexes with Mes*PCl_2_ gave M(ii) diphosphene complexes by P–Si bond cleavage and loss of ClSiMe_3_.^99*e*^

**Fig. 17:**
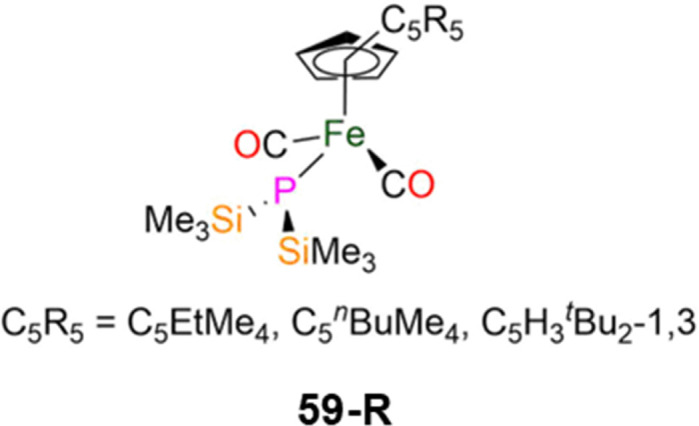
Group 8 P′′ complexes [Fe(C_5_R_5_)(CO)_2_(P′′)] (59-R; C_5_R_5_ = C_5_EtMe_4_, 59-EtMe_4_; C_5_^*n*^BuMe_4_, 59-^*n*^BuMe_4_; C_5_H_3_^*t*^Bu_2_-1,3, 59-1,3-^*t*^Bu_2_H_3_).

In 1992, Weber reported the synthesis of a series of Cp-substituted Fe(ii) P′′ complexes [Fe(C_5_R_5_)(CO)_2_(P′′)] (59-R; C_5_R_5_ = C_5_EtMe_4_, 59-EtMe_4_; C_5_^*n*^BuMe_4_, 59-^*n*^BuMe_4_; C_5_H_3_^*t*^Bu_2_-1,3, 59-1,3-^*t*^Bu_2_H_3_, Fig. 17) by the separate salt metathesis reactions of the parent monobromide complexes, [Fe(C_5_R_5_)(CO)_2_(Br)] (R_5_ = EtMe_4_, ^*n*^BuMe_4_, 1,3-^*t*^Bu_2_H_3_) with LiP′′.^99*f*^ The syntheses of three analogous Ru(ii) P′′ complexes [Ru(C_5_R_5_)(CO)_2_(P′′)] (R_5_ = 1,2,4-^i^Pr_3_H_2_, 1,3-^*t*^Bu_2_H_3_, 1,3-(SiMe_3_)_2_H_3_) were reported by Weber in 1994 but again these complexes were not structurally authenticated.^99*g*^ We note that only 59-EtMe_4_ was structurally characterised by single crystal XRD in the former paper,^99*f*^ thus comparative discussions of 59-R focus on NMR and IR spectroscopy. The Fe–P distance in 59-EtMe_4_ is 2.359(3) Å and in the solid state the phosphorus atom has a pyramidal geometry (329.2(2)°), with the trimethylsilyl groups eclipsed with respect to the CO ligands. The ^31^P{^1^H} NMR spectra of 59-R exhibit singlets at −219.1 ppm (59-EtMe_4_), −218.6 ppm (59-^*n*^BuMe_4_) and −266.4 ppm (59-1,3-^*t*^Bu_2_H_3_), whilst the IR spectra contain CO stretching modes at 1986 and 1939 cm^−1^ (59-EtMe_4_), 1988 and 1935 cm^−1^ (59-^*n*^BuMe_4_), and 1995 and 1944 cm^−1^ (59-1,3-^*t*^Bu_2_H_3_). It was deduced that a reduced donor strength for the 1,3-bis-*tert*-butyl-substituted C_5_R_5_ ligand gives rise to weaker M–C π-backbonding and therefore stronger CO bonding in 59-1,3-^*t*^Bu_2_H_3_. Finally, Weber reported that the separate reactions of 59-R or related Fe(ii) and Ru(ii) P′′ complexes with Mes*PCl_2_ gave M(ii) diphosphene complexes,^99*f*,*g*^ analogously to the reactions described above.^99*e*^

#### 2.3.6 Group 10 complexes

**Fig. 18:**
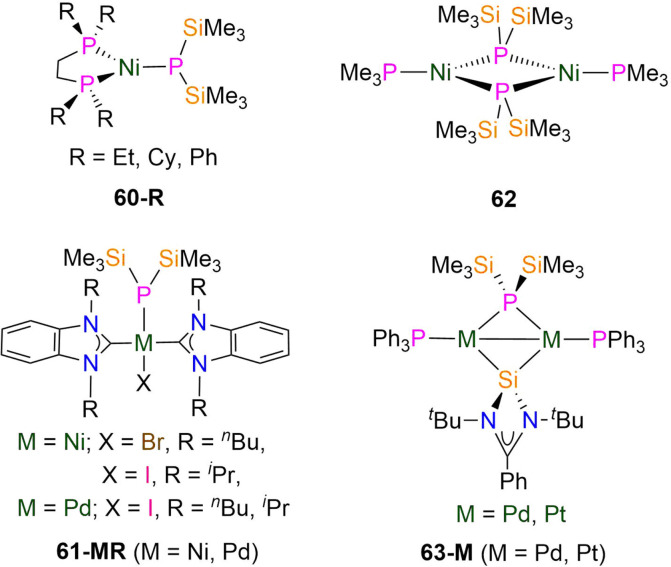
Group 10 P′′ complexes [Ni(κ^2^-drpe)(P′′)] (60-R; drpe = R_2_PCH_2_CH_2_PR_2_, R = Et, Cy, Ph), [M(X)(R_2_-bimy)_2_(P′′)] (61-MR. M = Ni, X = Br, R = ^*n*^Bu, 61-Ni^*n*^Bu; M = Ni, X = I, R = ^i^Pr, 61-Ni^i^Pr; M = Pd, X = I, R = ^*n*^Bu, 61-Pd^*n*^Bu; ^i^Pr, 61-Pd^i^Pr; bimy = benzimidazole-2-ylidene), [Ni(PMe_3_)(μ-P′′)]_2_ (62), [{M(PPh_3_)}_2_(P′′){Si[N(^*t*^Bu)]_2_CPh}] (63-M; M = Pd, Pt).

In 1987 Schäfer reported the synthesis of a family of monomeric Ni(ii) P′′ complexes, [Ni(κ^2^-drpe)(P′′)] (60-R; drpe = R_2_PCH_2_CH_2_PR_2_, R = Et, Cy, Ph, Fig. 18), by the separate salt metathesis reactions of two equivalents of LiP′′ with [Ni(κ^2^-drpe)(Cl)_2_] (R = Et, 60-NiEt; Cy, 60-NiCy; Ph, 60-NiPh); 60-NiCy was the only complex in this series to be structurally characterised.^100^ The intermediate mono-substituted complexes, [Ni(κ^2^-drpe)(P′′)(Cl)], were observed by *in situ* NMR spectroscopy but could not be isolated.^100^ The Ni atom in 60-NiCy is three-coordinate and nearly planar, with the P′′ phosphorus atom exhibiting a pyramidal geometry and a Ni–P bond length of 2.225(2) Å, which is slightly longer than the corresponding Ni–P distances of the coordinated diphosphine (2.192(2) and 2.202(2) Å).^100^ In 2015 Corrigan synthesised the Ni(ii) and Pd(ii) complexes, [M(X)(R_2_-bimy)_2_(P′′)] (61-MR. M = Ni, X = Br, R = ^*n*^Bu, 61-Ni^*n*^Bu; M = Ni, X = I, R = ^i^Pr, 61-Ni^i^Pr; M = Pd, X = I, R = ^*n*^Bu, 61-Pd^*n*^Bu; ^i^Pr, 61-Pd^i^Pr; bimy = benzimidazole-2-ylidene, Fig. 18) by the separate reactions of parent [MX_2_(R_2_-bimy)_2_] with LiP′′ in THF at 60 °C; the high reaction temperature was required due to the poor solubility of the group 10 metal starting materials.^101^ The ^i^Pr-substituted complexes, 61-Ni^i^Pr and 61-Pd^i^Pr, were found to undergo P–Si bond cleavage in the presence of benzoyl chloride to give the respective complexes [M(i)(^i^Pr_2_-bimy)_2_{P[C(O)Ph]_2_}] (M = Ni, Pd).^101^ Only the Pd analogues, 61-Pd^*n*^Bu and 61-Pd^i^Pr, were structurally characterised, and were both found to exhibit square-planar geometries at the d^8^ Pd(ii) centres as expected.^101^ The NHC ligands in 61-Pd^i^Pr cause a more distorted pyramidal geometry at the P atom (Si–P–Si: 98.53(6)°) compared to 61-Pd^*n*^Bu (Si–P–Si: 100.68(9)°);^101^ this difference was attributed to the larger steric requirements of ^i^Pr *vs*. ^*n*^Bu groups, as the latter are able to fold away to a greater extent to allow for the steric requirements of P′′.^101^ The Pd–P bond lengths vary slightly between the two complexes (2.3648(17) Å, 61-Pd^*n*^Bu; 2.3442(12) Å, 61-Pd^i^Pr) and there is a greater distortion from linearity of the C–Pd–C (171.41(13)°) and I–Pd–P (166.42(3)°) angles in 61-Pd^i^Pr compared to 61-Pd^*n*^Bu (176.6(2)° and 174.55(4)°, respectively).^101^ It was noted that the two ^*n*^Bu_2_-bimy ligands of 61-Pd^*n*^Bu are twisted at 26.8° with respect to each other.^101^

In 1982, Schäfer reported the synthesis of the dimeric Ni(i) P′′ complex [Ni(PMe_3_)(μ-P′′)]_2_ (62, Fig. 18) by the decomposition of [Ni(PMe_3_)_2_(P′′)_2_] at room temperature to form both [Ni(PMe_3_)_2_{μ-P(SiMe_3_)}_2_] and [Ni(PMe_3_)(μ-P′′)]_2_.^102^ In the solid state 62 exhibits a planar central Ni_2_P_2_ ring in which each Ni atom is three-coordinate and bound to two bridging P′′ and one terminal trimethylphosphine.^102^ The Ni–P bond lengths within the central core are 2.186(1) Å and the Ni–P distances for the terminal phosphines are 2.129(2) Å; the Ni⋯Ni separation is 2.381(1) Å.^102^ In 2013 Inoue reported the synthesis of the dinuclear M(i) P′′ complexes [{M(PPh_3_)}_2_(P′′){Si[N(^*t*^Bu)]_2_CPh}] (63-M; M = Pd, Pt; Fig. 18); 63-Pt was characterised by both NMR spectroscopy and single crystal XRD, whereas the formation of 63-Pd was determined by NMR spectroscopy and high resolution mass (HRMS) spectrometry only.^103^ The ^1^H NMR spectra of 63-M exhibit singlets (0.92 ppm, 63-Pt; 0.86 ppm, 63-Pd) for the ^*t*^Bu groups, as well as a doublet (0.46 ppm, ^3^*J*_PH_ = 5.2 Hz, 63-Pt; 0.54 ppm, ^3^*J*_PH_ = 5.2 Hz, 63-Pd) for the trimethylsilyl groups. In the ^31^P{^1^H} NMR spectrum of 63-Pt a doublet at 56.0 ppm (^1^*J*_PtP_ = 5137 Hz and ^2^*J*_PtP_ = 60.5 Hz, 33.8% abundant ^195^Pt *I* = ½) and a triplet at −94.6 ppm (^1^*J*_PtP_ = 1633 Hz) are observed that respectively correspond to PPh_3_ and P′′, and these are seen at 41.2 and −163.4 ppm, respectively, for 63-Pd.^103^ The Pt–Pt bond length of 63-Pt (2.6466(5) Å) falls within the reported range of Pt–Pt bonds (2.4059(6)-2.7183(10) Å),^9^ whilst the P′′ Pt–P bond lengths (2.360(2) and 2.367(2) Å) are longer than the corresponding distances for the coordinated phosphines of 2.222(2) and 2.213(2) Å.^103^

#### 2.3.7 Group 11 complexes

**Fig. 19:**
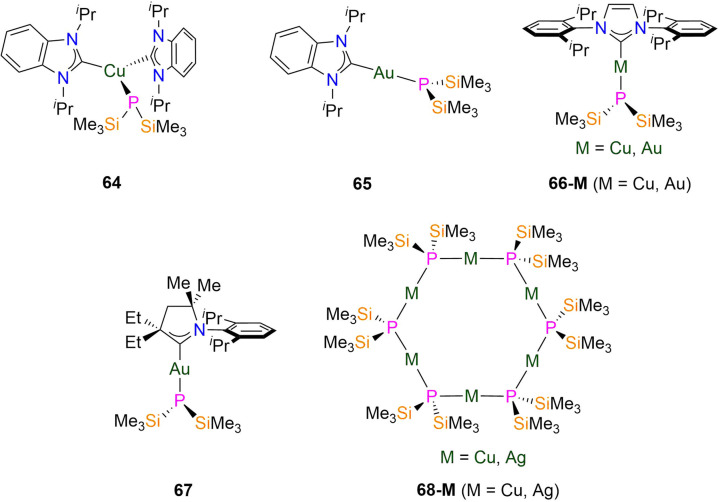
Group 11 P′′ complexes [Cu(^i^Pr_2_-bimy)_2_(P′′)] 64 (^i^Pr_2_-bimy = 1,3-di-isopropylbenzimidazole-2-ylidene), [Au(^i^Pr_2_-bimy)(P′′)] (65),; [M(IPr)(P′′)] (66-M; M = Cu, Au), IPr = (1,3-bis(2,6-diisopropylphenyl)-2-ylidene), [Au(CAAC^MeEt^)(P′′)] (67, CAAC^MeEt^ = dimethyl-diethyl-cyclic(alkyl)(amino)carbene), [M(μ-P′′)]_6_ (68-M; M = Cu, Ag).

N-Heterocyclic carbene (NHC) ligands have been used as supporting ligands in d-transition metal P′′ complexes in an effort to provide stable complexes that are suitable for long term storage.^104^ Between 2015–2016 Corrigan reported the syntheses of three types of group 11 P′′ complexes: [Cu(^i^Pr_2_-bimy)_2_(P′′)] (64, ^i^Pr_2_-bimy = 1,3-diisopropylbenzimidazole-2-ylidene) by the reaction of [Cu(^i^Pr_2_-bimy)_2_(OAc)] with P(SiMe_3_)_3_, [Au(^i^Pr_2_-bimy)(P′′)] (65) by the reaction of [Au(^i^Pr_2_-bimy)(Cl)] with P(SiMe_3_)_3_, and [M(IPr)(P′′)] (66-M; M = Cu, Au, IPr = 1,3-bis(2,6-diisopropylphenyl)-2-ylidene) by the reaction of [Cu(IPr)(OAc)] or [Au(IPr)(Cl)] with P(SiMe_3_)_3_ (Fig. 19); complex 65 was not structurally authenticated.^104,105^ At the time of publication 64 and 66-M provided some of the first examples of structurally authenticated terminal d-transition metal P′′ complexes. The Cu centre in 64 exhibits a distorted trigonal planar geometry and the phosphorus atom is trigonal pyramidal; together with a long Cu–P bond length (2.291(7) Å); this demonstrates that the phosphorus lone pair does not contribute to the M–P bonding.^104^ Complex 66-Cu exhibits a shorter Cu–P bond length of 2.1913(15) Å and a near-linear geometry (C–Cu–P: 173.39(17)°), with the phosphorus atom being distorted trigonal pyramidal;^104^ similarly, 66-Au has a near-linear geometry (C–Au–P: 175.74(4)°) and a distorted trigonal pyramidal phosphorus atom, though the Au–P bond length in 66-Au is longer (2.3174(10) Å).^105^ The ^1^H NMR spectrum of 64 confirms that the solid state structure is maintained in solution as there is a 2 : 1 ratio of ^i^Pr_2_-bimy: P′′, together with one resonance in the ^31^P{^1^H} NMR spectrum at −261 ppm; the analogous signal for 65 is at *δ*_P_ = −235 ppm.^104,105^ The ^1^H NMR spectrum of 66-Cu showed that the solid-state structure was retained in solution due to the 1 : 1 ratio of IPr: P′′ ligands. The ^31^P{^1^H} NMR spectra of 66-M each show one resonance, at −268 ppm for the Cu analogue and −235.7 ppm for the Au congener.^104,105^ In 2023 Liu reported the synthesis of [Au(CAAC^MeEt^)(P′′)] (67; CAAC^MeEt^ = dimethyl-diethyl-cyclic(alkyl)(amino)carbene, Fig. 19) by the reaction of [Au(CAAC^MeEt^){P(Ph)_2_}] with KP′′.^106^ This synthetic route is remarkable as it involves Au–P bond cleavage occurring, despite the strong covalency between Au and P due to relativistic effects which result in Au having the highest electronegativity of any d-transition metal (Pauling *χ* = 2.54). As with 66-M, 67 exhibits a near-linear geometry (C–Au–P: 174.45(5)°) with a distorted trigonal pyramidal geometry about the phosphorus atom and an Au–P bond length of 2.3253(5) Å. Complex 67 has one resonance in its ^31^P{^1^H} NMR spectrum at −233.1 ppm.^106^

Group 11 metal P′′ cluster complexes have been synthesised in order to explore their potential as precursors for semiconductor materials. The group 11 hexameric cyclic complexes [M(μ-P′′)]_6_ (68-M; M = Cu, Ag; Fig. 19) were synthesised in 2015 by Corrigan by the separate reactions of parent polymeric [M(S^*t*^Bu)]_*n*_ with equimolar P(SiMe_3_)_3_*via* the cleavage of P–Si and M–S bonds and the formation of ^*t*^BuSSiMe_3_ and 68-M.^104^ In the solid state the P atoms of 68-M form the corners of a planar hexagonal structure, and the two-coordinate metal centres form the sides.^104^ The M–P bond lengths range from 2.2043(16)–2.2094(15) Å for 68-Cu and 2.393(1)–2.411(2) Å for 68-Ag, and the P–M–P angles deviate slightly from linearity in both complexes (∼178°, 68-Cu; ∼177°, 68-Ag).^104^ The phosphorus atoms in 68-M exhibit distorted tetrahedral geometries such that the trimethylsilyl groups are equally arranged above and below the plane of the ring. It was noted that the voids above and below the centre of the ring are occupied by THF lattice solvent molecules, which is believed to be important for the formation of the cyclic structures, and this sensitivity is in line with the loss of solvent leading to discolouration of solutions of 68-M.^104^ NMR spectroscopy showed that the symmetrical structures are retained in solution for both complexes, with only one resonance in each ^1^H NMR spectrum at 0.50 ppm (68-Cu) and 0.48 ppm (68-Ag), and each ^31^P{^1^H} NMR spectrum at −149 ppm (68-Cu) and −236 ppm (68-Ag).^104^

#### 2.3.8 Group 12 complexes

**Fig. 20:**
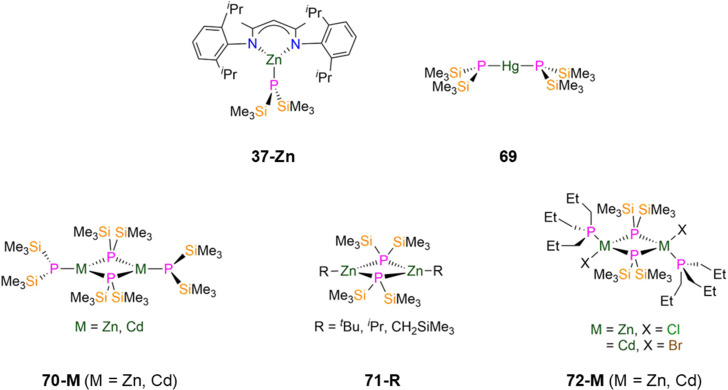
Monomeric and dimeric group 12 P′′ complexes [Zn(^Dipp^Nacnac)(P′′)] (37-Zn; ^Dipp^Nacnac = CH{(CH_3_)_2_CN(Dipp)}_2_; Dipp = C_6_H_3_^i^Pr_2_-2,6), [Hg(P′′)_2_] (69), [M(P′′)(μ-P′′)]_2_ (70-M, M = Zn, Cd), [Zn(R)(μ-P′′)]_2_ (71-R, R = ^*t*^Bu, ^i^Pr, CH_2_SiMe_3_), [M(P^*n*^Pr_3_)(X)(μ-P′′)]_2_ (72-Zn: M = Zn, X = Cl; 72-Cd: M = Cd, X = Br).

In 2024, Weigend and Hänisch synthesised the monomeric Zn(ii) complex [Zn(^Dipp^Nacnac)(P′′)] (37-Zn, Fig. 20) in an analogous manner to that discussed earlier for 37-M (M = Cr, Mn).^140^ The Zn centre of 37-Zn has a distorted trigonal pyramidal geometry with a Zn–P bond length of 2.2728(3) Å, which compares well to other literature compounds such as [Zn(P′′)(μ-P′′)]_2_ (mean Zn–P_terminal_: 2.295(1) Å).^83,140^ The ^31^P{^1^H} NMR spectrum of 37-Zn has one resonance at −288.85 ppm, with satellites observed due to coupling to ^29^Si nuclei (^1^*J*_PSi_ = 28.5 Hz).^140^ In 1993 Buhro synthesised a monomeric Hg(ii) P′′ complex [Hg(P′′)_2_] (69, Fig. 20) by the reaction of [Hg(N′′)_2_] with HP′′.^83^ Complex 69 displays a near-linear geometry in the solid state (P–Hg–P = 175.8°), and the phosphorus atoms have pyramidal geometries.^83^ The monomers of 69 are oriented in the lattice such that long-range dimers are formed with two bridging and two terminal P′′. However, the resultant bridging Hg⋯P interactions are only slightly shorter (3.246(1) Å) than the sum of the van der Waals radii of Hg and P (3.35 Å). This interaction can be seen as a weak dative bond, which leads to the Hg–P bond lengths being nearly equal to one another (2.402(1) and 2.410(1) Å).^83^ The NMR spectra of 69 indicate that a monomeric form dominates in solution, with one resonance seen in both ^1^H (0.36 ppm) and ^31^P{^1^H} (−162.0 ppm) NMR spectra.^83^

Many dimeric group 12 complexes have been reported with bridging P′′, with varying terminal ligands such as alkyls, aryls, halides, phosphines and phosphides. In 1993 Buhro synthesised the homoleptic M(ii) P′′ complexes [M(P′′)(μ-P′′)]_2_ (70-M, M = Zn, Cd, Fig. 20) by the protonolysis reaction of [M(N′′)_2_] (M = Zn, Cd) with HP′′.^83^ Single crystal XRD studies of 70-M showed that each metal in the dimer has a distorted trigonal planar geometry and is bound by two bridging and one terminal P′′, with the terminal phosphorus atom being pyramidal; the distorted rectangular M_2_P_2_ cores in 70-M have bridging M–P–M angles which are closer to 90° rather than the usual 120°. The M–P_bridging_ (2.419(1) and 2.421(1) Å for 70-Zn; 2.575(1) and 2.612(1) Å for 70-Cd) and M–P_terminal_ (2.295(1) Å, 70-Zn; 2.459(1) Å, 70-Cd) bond lengths also lie within the expected range for bridging and terminal Zn–P and Cd–P bonds, respectively (CSD: Zn–P (2.212(3)–2.9159(7) Å), Cd–P (2.2379(8)–2.800(6) Å)).^9,83^ Both complexes exhibit fluxional properties in solution, with low temperature NMR data (230 K) consistent with the presence of the dimer, as evidenced by two resonances in the ^31^P{^1^H} NMR spectra for bridging and terminal phosphides (−183.0 ppm, P_bridging_, −237.3 ppm P_terminal_, 70-Zn; −180.1 ppm, P_bridging_, −229.5 ppm, P_terminal_, 70-Cd).^83^ Upon conducting high temperature NMR experiments, line broadening and coalescence of these two resonances is seen for both complexes, which was assigned to bridging-to-terminal site exchange with a calculated barrier of Δ*G*^‡^_360_ = 14.3(2) kcal mol^−1^ (70-Zn) and Δ*G*^‡^_321_ = 12.7(6) kcal mol^−1^ (70-Cd).^83^ The mechanism of this exchange was proposed to be dissociative where the dimers dissociate into the respective monomers and reassociate, or that one of the M–P_bridging_ bonds is broken, the remaining M–P_bridging_ bond rotates, and then the cleaved M–P_bridging_ bond reforms to give the exchanged dimer.^83^

The synthesis of the dimeric Zn(ii) P′′ complexes [Zn(R)(μ-P′′)]_2_ (71-R; R = ^*t*^Bu, ^i^Pr, CH_2_SiMe_3_; Fig. 20) was reported in 1995 by Westerhausen by the reaction of parent ZnR_2_ (R = ^*t*^Bu, ^i^Pr, CH_2_SiMe_3_) with HP′′; however 71-^*t*^Bu was not structurally characterised.^107^ Complexes 71-^i^Pr and 71-CH_2_SiMe_3_ are dimers that consist of two trigonal planar Zn centres with bridging P′′ and terminal alkyl groups, with nearly planar Zn_2_P_2_ cores. The Zn–P bond lengths are 2.405(3) and 2.416(3) Å for 71-^i^Pr and 2.394(1) and 2.436(1) Å for 71-CH_2_SiMe_3_.^107^^31^P{^1^H} NMR spectroscopy showed one resonance for each complex, at −216.3 ppm (71-^i^Pr), −205.8 ppm (71-CH_2_SiMe_3_) and −216.7 ppm (71-^*t*^Bu).^107^ Dimeric group 12 metal P′′ complexes [M(P^*n*^Pr_3_)(X)(μ-P′′)]_2_ (72-Zn: M = Zn, X = Cl; 72-Cd: M = Cd, X = Br; Fig. 20) featuring both a terminal phosphine and halide were synthesised in 1996 by Fenske *via* the separate reactions of MX_2_ (M = Zn, X = Cl; M = Cd, X = Br) with P(SiMe_3_)_3_ in the presence of P^*n*^Pr_3_.^108^ Both complexes exhibit distorted tetrahedral metal centres in the solid state, with the central M_2_P_2_ rings showing planar geometries with typical M–P_bridging_ (2.426(1) and 2.447(1) Å for 72-Zn; 2.571(1) and 2.609(1) Å for 72-Cd) and M–P_terminal_ (2.408(1) and 2.430(1) Å for 72-Zn; 2.584(1) and 2.571(1) Å for 72-Cd) bond distances.^108^

**Fig. 21:**
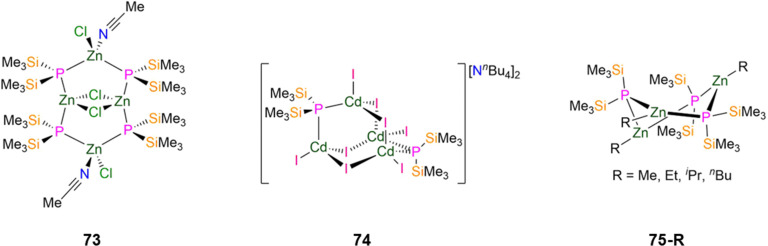
Group 12 P′′ cluster complexes [{Zn(Cl)(MeCN)(μ-P′′)_2_}_2_{Zn(μ-Cl)}_2_] (73), [N^*n*^Bu_4_]_2_[Cd_4_(i)_8_(P′′)_2_] (74), [Zn(R)(P′′)]_3_ (75-R; R = Me, Et, ^i^Pr, ^*n*^Bu).

In 1996, Fenske reported the synthesis of a Zn(ii) P′′ cluster complex, [{Zn(Cl)(MeCN)(μ-P′′)_2_}_2_{Zn(μ-Cl)}_2_] (73, Fig. 21), by the reaction of ZnCl_2_ with equimolar P(SiMe_3_)_3_ in MeCN.^108^ Single crystal XRD revealed that the four Zn and four P atoms form a Zn_4_P_4_ eight membered ring, with two chlorides bridging two of the Zn centres to give a Zn_2_Cl_2_ core. The two Zn atoms that are not involved in the central four-membered ring are distorted tetrahedral and coordinated by two bridging P′′ (mean Zn–P: 2.405(2) Å), a terminal chloride and MeCN, whereas the coordination spheres of the other two Zn atoms are completed by bridging P′′, with a shorter mean Zn–P bond length (2.343(2) Å) giving much more strongly distorted tetrahedra (P–Zn–P: 153.88(4)°). Solutions of 73 decompose upon warming to room temperature, precluding the collection of NMR spectroscopic data that could be confidently assigned.^108^ In the same report a Cd(ii) cluster complex, [N^*n*^Bu_4_]_2_[Cd_4_(i)_8_(P′′)_2_] (74, Fig. 21), was formed from the reaction of [Cd(i)_2_{P(SiMe_3_)_3_}]_2_ with N^*n*^Bu_4_I, with ISiMe_3_ eliminated as a byproduct.^108^ Complex 74 consists of four C atoms with four bridging iodides and two bridging P′′ to yield an adamantane-like skeleton; each distorted tetrahedral Cd atom also bears one terminal iodide.^108^ The mean Cd–P bond distance is 2.507(7) Å, which is shorter than those found for other Cd–P_bridging_ bonds in 70-Cd (2.612(1) Å) and 72-Cd (2.609(1) Å).^83,108^ The trimeric Zn(ii) P′′ complexes [Zn(R)(P′′)]_3_ (75-R; R = Me, Et, ^i^Pr, ^*n*^Bu, Fig. 21) were reported in 1994 by Westerhausen, *via* the addition of ZnR_2_ to HP′′. Single crystal XRD showed that these complexes contain six-membered Zn_3_P_3_ rings in twisted boat conformations, with the three Zn atoms having near trigonal planar geometries with terminal alkyl groups, and the phosphorus atoms showing distorted tetrahedral geometries; the solid-state structure of 75-Et was not reported.^107^ In solution this series of complexes were found to exhibit equilibria with the dimeric complexes discussed earlier in this section (71-R), with the larger alkyl groups favouring the formation of dimers.^107^ For complex 75-Me, two of the phosphorus atoms are situated 0.525 Å and 0.857 Å above and below the plane, respectively, whereas in 75-^*n*^Bu these two phosphorus atoms are located more symmetrically at ±0.640 Å.^107^ The mean Zn–P bond lengths of 2.390(3) Å (75-Me), 2.408(6) Å (75-^i^Pr) and 2.388(4) Å (75-^*n*^Bu) fall within the expected range for bridging Zn–P bonds (2.245(4)–2.8049(7) Å).^9,107^ Chemical shifts in the ^31^P{^1^H} NMR spectra for the trimeric complexes of −246.8 ppm (75-Me), −246.9 ppm (75-Et), −243.6 ppm (75-^i^Pr), −246.0 ppm (75-^*n*^Bu) are shifted to lower chemical shifts than the related dimers, which are seen at–216.3 ppm (71-^i^Pr) and −216.7 ppm (71-^*t*^Bu).^107^

#### 2.3.9 Heterometallic complexes

**Fig. 22:**
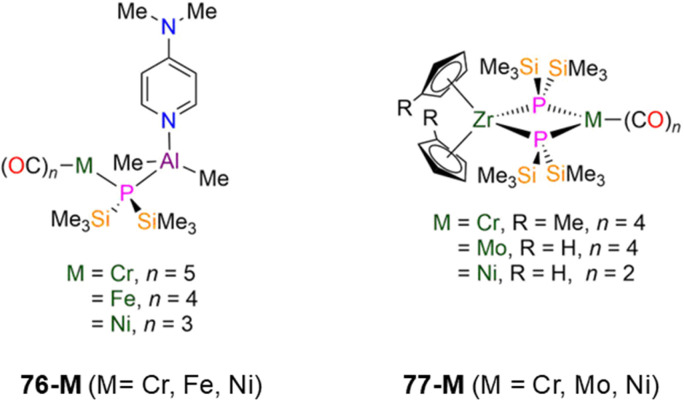
Heterometallic P′′ complexes [M(CO)_*n*_(μ-P′′){Al(dmap)(Me_2_)}] (76-M; M = Cr, *n* = 5, 76-Cr; M = Fe, *n* = 4, 76-Fe; M = Ni, *n* = 3, 76-Ni), [Zr(Cp)_2_(μ-P′′)_2_M(CO)_*n*_] (77-M; M = Mo, *n* = 4, 77-Mo; M = Ni, *n* = 2, 77-Ni).

To date, all examples of heterometallic d-block P′′ complexes are also bound by carbon monoxide. From 2001–2002, Schulz reported the separate reactions of [Al(Me)_2_(P′′)(dmap)] (17-Al, see Section 2.2.1) with the d-transition metal carbonyl complexes [Cr(NMe_3_)(CO)_5_], [Fe_3_(CO)_12_] and [Ni(CO)_4_] to give the respective M(0) P′′-bridged complexes, [M(CO)_*n*_(μ-P′′){Al(dmap)(Me)_2_}] (M = Cr, *n* = 5, 76-Cr; M = Fe, *n* = 4, 76-Fe; M = Ni, *n* = 3, 76-Ni, Fig. 22).^39,109^ Complexes 76-M exhibit staggered conformations, with N–Al–P–M torsion angles of 36.9(1)° for 76-Cr and 34.1(1)° for 76-Fe; 76-Ni shows a range of N–Al–P–Ni torsion angles from 21.60(13)–88.05(13)° as there are six independent molecules in the unit cell.^39,109^ The M–P bond lengths are 2.528(1) Å (76-Cr), 2.377(1) Å (76-Fe) and 2.315(2) Å (76-Ni), with the former two being the longest reported examples of such bonds in P–M(CO)_*n*_ moieties for these metals at the time of publication.^39,109^ The long Al–P bonds and short Al–N bonds in 76-M, combined with their long P–M(CO)_*n*_ bonds, indicate that there is some weak M → P π-backbonding within these systems.^39,109^ Dimeric heterobimetallic P′′ complexes were separately reported by Hey-Hawkins and Eisen in order to study the cooperative reactivity between an early and late d-transition metal for their potential in the heterolytic bond cleavage of polar substrates.^111^ In 1995–1996, the separate reactions of the Zr(iv) complex, [Zr(Cp)_2_(P′′)_2_] (77-M, Fig. 22), with [Ni(CO)_4_] or [Mo(CO)_4_(NBD)] (NBD = norbornadiene) gave [Zr(Cp)_2_(μ-P′′)_2_M(CO)_*n*_] (M = Mo, *n* = 4, 77-Mo; M = Ni, *n* = 2, 77-Ni);^110,111^ a similar complex [Zr(C_5_H_4_Me)_2_(μ-P′′)_2_Cr(CO)_*n*_] (77-Cr) has also been synthesised *via* analogous methods from [Cr(CO)_4_(NBD)] and 51.^110^ The central ZrP_2_M rings in these complexes are slightly puckered, with equivalent Zr–P bond lengths of 2.654(4) and 2.657(4) Å (77-Cr), 2.6711(9) Å and 2.6585(7) Å (77-Mo) and 2.655(1), 2.652(1) Å (77-Ni) as well as equivalent M–P bond lengths of 2.513(4) and 2.502(4) Å (77-Cr), 2.6263(7) and 2.6311(10) Å (77-Mo) and 2.264(1) and 2.266(1) Å (77-Ni).^110,111^ There are no Zr–M bonds in these complexes, with Zr⋯M distances of 3.414(3) Å (77-Cr), 3.459(3) Å (77-Mo) and 3.038(1) Å (77-Ni).^110,111^ The ^31^P{^1^H} NMR spectra each exhibit a single resonance at −38.4 ppm (77-Cr), −57.9 ppm (77-Mo), −42.1 ppm (77-Ni), which are all at higher frequencies than the starting material (−71.1 ppm, 48-Zr).^110,111^ Complexes 77-Ni and 77-Mo did not demonstrate any catalytic activity towards the polymerisation of ethylene. However, when these complexes were activated by a Lewis acid such as methylaluminoxane (MAO), polymerisation of ethylene occurred and was found to outperform cationic group 4 metallocene systems.^110,111^

### 2.4 f-Block P′′ complexes

#### 2.4.1 Lanthanides

**Fig. 23:**
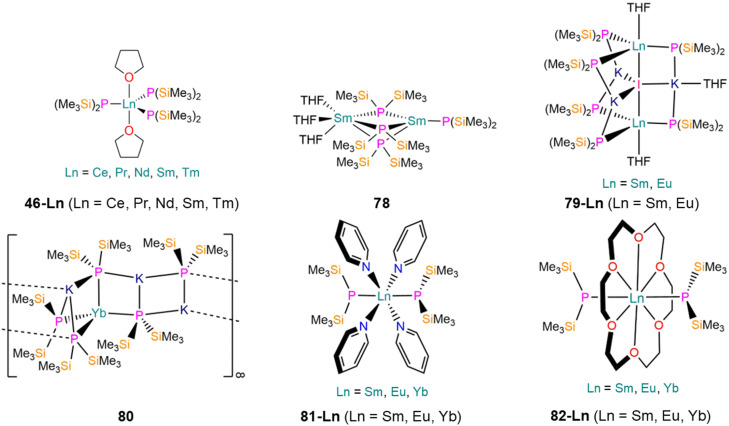
Ln P′′ complexes [Ln(P′′)_3_(THF)_2_] (46-Ln; Ln = Ce, Pr, Nd, Sm, Tm), [Sm(P′′)(μ-P′′)_3_Sm(THF)_3_] (78), [{Ln(P′′)_3_(THF)}_2_(μ-I)K_3_(THF)] (79-Ln; Ln = Sm, Eu), [KYb(P′′)_3_{μ-K(P′′)}_2_]_∞_ (80), *trans*-[Ln(P′′)_2_(py)_4_] (81-Ln; Ln = Sm, Eu, Yb), [Ln(P′′)_2_(18-crown-6)] (82-Ln; Ln = Sm, Eu, Yb).

Rabe reported the first Ln P′′ complexes, [Ln(P′′)_3_(THF)_2_] (46-Ln, Fig. 23) (Ln = Nd, Tm) in 1995, which at the time were the first structurally characterised Ln tris-phosphide complexes.^112,113^ Later, in 2024, Mills and co-workers reported further examples in this series for Ln = La, Ce, Pr and Sm.^89^ All complexes were prepared by salt metathesis reactions of the respective [Ln(i)_3_(THF)_3.5_] starting material with three equivalents of KP′′. Single crystal XRD showed that 46-Ln have distorted trigonal bipyramidal geometries, with three equatorial P′′ and two axial THF molecules, with the O–Ln–O angles showing a small deviation from linearity: 175.6(6) (46-Ce), 175.58(13) (46-Pr), 175.68(9) (46-Nd), 175.36(13) (46-Sm) and 172.06(14)° (46-Tm).^89,112,113^ Two of the equatorial P′′ in 46-Ln show pyramidal geometries about the phosphorus atom, whereas the third P′′ exhibits a planar geometry.^89,112,113^ The mean Ln–P bond lengths (2.849(3) (46-Ce), 2.837(3) (46-Pr), 2.818(2) (46-Nd), 2.789(3) (46-Sm) and 2.707(16) (46-Tm) Å) can be compared to the mean Ln–N distances in the Ln(iii) N′′ complexes [Ln(N′′)_3_] (Ln = Nd, 2.244(16); Yb, 2.149(12) Å);^141–143^ the structure of [Tm(N′′)_3_] has not been reported, but the six-coordinate ionic radius of Yb(iii) (0.868 Å) is similar to that of Tm(iii) (0.880 Å).^123^ The differences in solvation between 46-Ln and [Ln(N′′)_3_] can be attributed to the Ln–P bonds in the former being longer that the Ln–N bonds in the latter leading to a reduced steric saturation of the Ln coordination sphere.^112,113^ Comparisons may also be made between 46-Ln and the structurally analogous solvated Ln(iii) tris-amide complexes [Ln{N(SiMe_2_H)_2_}_3_(THF)_2_] (Ln = Nd, Nd–N = 2.344(9) Å, O–Nd–O = 163.18(15)°; Ln = Yb, Yb–N = 2.224(8) Å, O–Yb–O = 160.50(14)°).^114,144^ The solution ^31^P{^1^H} NMR spectra of 46-Ln (Ln = Ce, Pr, Nd, Sm) exhibited broad and paramagnetically shifted singlets at 616.7 (46-Ce), 1894.2 (46-Pr), 2570.3 (46-Nd) and −259.2 (46-Sm) ppm with no resonances being observed in ^13^C{^1^H} and ^31^P{^1^H} NMR spectra that could be assigned to 46-Tm.^89,112,113^ Solid state ^31^P MAS NMR spectroscopy of 46-Ln (Ln = Ce–Sm) revealed two components in their spectra in a 2 : 1 ratio, with the major component assigned to pyramidal and the minor component to planar P environments.^89^ This is in contrast to the one resonance observed in the solution ^31^P{^1^H} NMR spectra of 46-Ln due to dynamic processes and broad resonances.^89^

In 1996, Rabe reported the synthesis of the bimetallic Sm(ii) complex [Sm(P′′)(μ-P′′)_3_Sm(THF)_3_] (78, Fig. 23) by the salt metathesis reaction of two equivalents of KP′′ with SmI_2_(THF)_2_ in THF at room temperature, analogously to the preparation of 14 discussed in Section 2.1.1.^125^ Single crystal XRD showed that 78 features one four-coordinate Sm ion with one terminal and three bridging P′′, and one six-coordinate Sm ion with three bridging P′′ and three bound THF. The N′′ analogue of 78, [Sm(N′′)_2_(THF)_2_],^145^ is monometallic, following a general trend that higher nuclearities tend to be observed for P′′ complexes compared to their N′′ counterparts.^146^ The solid state structure of 78 differs from those of the six-coordinate monometallic Ln(ii) bis-(diphenyl)phosphide complexes, [Ln(PPh_2_)_2_(THF)_4_] (Ln = Sm, Yb) and [Sm(PPh_2_)_2_(*N*-methylimidazole)_4_],^147^ and bears more resemblance to the solvent-free Eu(ii) N′′ ‘ate‘ complex, [Eu(N′′)(μ-N′′)_2_Na].^148^ The terminal Sm–P bond length of 78 (3.027(3) Å) is similar to the mean bridging Sm–P distances (3.039(5) Å), and all are shorter than those reported for six-coordinate [Sm(η^1^-dibenzophospholyl)_2_(THF)_4_] (3.1908(6) Å),^148^ [Sm(PPh_2_)_2_(*N*-methylimidazole)_4_] (3.139(3) Å),^147^ and [Sm(η^5^-C_8_H_4_PMe_2_-2,3)_2_(THF)_2_] (3.0775(1) Å).^149^ VT ^1^H NMR spectra of 78 were collected in both *d*_8_-toluene and *d*_8_-THF in order to determine its structure in solution, but these studies were inconclusive. In *d*_8_-THF at 293 K there is a singlet at 0.42 ppm (*ν*_1/2_ = 10 Hz), and at 173 K this becomes two singlets at 3.94 ppm (*ν*_1/2_ = 1 6 Hz) and −0.01 ppm (*ν*_1/2_ = 8 Hz). In *d*_8_-toluene at 293 K there is one singlet at 0.99 ppm (*ν*_1/2_ = 20 Hz), but at 193 K seven broad resonances were observed at 13.60, 8.50, 6.10, 3.80, 3.10, −0.54 and −2.93 ppm.^125^

In 2024, Mills and co-workers showed that the addition of two or three equivalents of KP′′ to [LnI_2_(THF)_2_] (Ln = Sm, Eu, Yb) in Et_2_O at −78 °C gave the dimeric Ln(ii) P′′ complexes [{Ln(P′′)_3_(THF)}_2_(μ-I)K_3_(THF)] (79-Ln; Ln = Sm, Eu) and polymeric [KYb(P′′)_3_{μ-K(P′′)}_2_]_∞_ (80), Fig. 23.^115^ The addition of pyridine to either 79-Ln or 80 at room temperature gave the monomeric pyridine-solvated Ln(ii) P′′ complexes *trans*-[Ln(P′′)_2_(py)_4_] (81-Ln; Ln = Sm, Eu, Yb), whereas the addition of 18-crown-6 to 79-Ln and 80 at room temperature afforded [Ln(P′′)_2_(18-crown-6)] (82-Ln; Ln = Sm, Eu, Yb), Fig. 23.^115^ The dimeric Ln(ii) ‘ate’ complexes 79-Ln consist of two {KLn(P′′)_3_(THF)} fragments with KI encapsulated between them. The Ln centres exhibit distorted trigonal bipyramidal geometries with three equatorial P′′, one axial THF and one axial iodide, which bridges the two fragments.^115^ The mean Ln–P bond lengths in 79-Ln (Ln = Sm, 3.033(7) Å; Eu, 3.038(7) Å) are shorter than the bridging Sm–P′′ bonds in 78 but are statistically equivalent to the terminal Sm–P′′ bond length of 78.^115,125^ Due to the paramagnetic nature of Sm(ii) and Eu(ii) ions, no resonances were observed in the ^31^P{^1^H} NMR spectra of 79-Ln.^115^ In the solid state 80 is a 1D coordination polymer with P′′ bridging Yb and K cations.^115^ The Yb centres are each coordinated by four P′′ and exhibit highly distorted tetrahedral geometries (P–Yb–P angles: 93.79(13)°, 98.4(2)°, 98.5(2)°, 105.94(14)°, 126.55(14)° and 125.8(2)°).^115^ The Yb–P bond lengths in 80 range between 2.837(7)–3.043(3) Å, with the mean Yb–P bond length (2.952(10) Å) being shorter than the Ln–P distances in 79-Ln due to the smaller ionic radius of Yb(ii) compared with Sm(ii) and Eu(ii).^115^ Despite Yb(ii) being a diamagnetic ion, it was noted that no resonances could be observed in the ^171^Yb{^1^H} NMR spectrum of 80. However, multiple resonances were observed in the ^1^H, ^13^C, ^29^Si DEPT90 and ^31^P{^1^H} NMR spectra. VT ^1^H and ^31^P{^1^H} NMR experiments between 213–323 K did not provide signals that could be confidently assigned to 80, which was attributed to rapid aggregation processes.^115^ Upon cooling, the major signal at *δ*_P_ = −219.0 ppm broadens further and two new signals form at *ca*. −211 and −243 ppm, but no ^171^Yb (14.3% abundant, *I* = ½) satellites could be assigned. It was therefore concluded that 80 converts into a complex mixture of aggregates in solution, which are in constant dynamic equilibria.^115^

In the solid state 81-Ln exhibit distorted octahedral geometries, with *trans*-configurations consisting of two axial P′′ and four equatorial pyridines.^115^ Complexes 81-Sm and 81-Eu are more distorted from the ideal octahedral geometry than 81-Yb, with the former exhibiting three N–Ln–N angles between 74.87(12)–77.75(14)° and a fourth of 133.3(2)° for 81-Sm and 131.81(12)° for 81-Eu, whereas for 81-Yb the range of N–Ln–N angles is much closer to ideal 90° (86.16(8)–92.09(7)°).^115^ This was attributed to the larger Sm(ii) and Eu(ii) ions allowing for two Me groups from P′′ ligands to form additional electrostatic interactions, with Ln⋯C distances of 3.809(5) Å (81-Sm) and 3.840(4) Å (81-Eu).^115^ Unlike for 80, all multinuclear NMR spectra could be assigned for the diamagnetic complex 81-Yb, though it was noted that drops of pyridine needed to be added to the sample in order to provide solution stability.^115^ The ^13^C{^1^H} NMR spectrum of 81-Yb exhibits silyl group resonances that are virtual triplets due to splitting by strongly coupled ^31^P nuclei (*δ*_C_ = 7.85 ppm, ^2^*J*_PC_ = 5.4 Hz).^115^ These higher order effects are also seen in the ^29^Si DEPT90 NMR spectrum (*δ*_Si_ = 1.58 ppm, ^1^*J*_PSi_ = 16 Hz).^115^ The ^31^P{^1^H} NMR spectrum of 81-Yb exhibits a resonance at −253.93 ppm, with satellites due to ^171^Yb nuclei with a ^1^*J*_YbP_ coupling constant of 925 Hz. ^147^ The ^171^Yb{^1^H} NMR spectrum of 81-Yb shows a triplet resonance at 1075.50 ppm, with the same ^1^*J*_YbP_ coupling constant as seen in the ^31^P{^1^H} NMR experiment.^115^

For complexes 82-Ln, distorted hexagonal bipyramidal geometries are observed, with mutually *trans*-P′′, and 18-crown-6 coordinated about the equatorial plane. Longer Ln–P bond lengths are observed for 82-Ln (82-Sm: 3.089(3) Å; 82-Eu: 3.086(6) Å; 82-Yb: 2.9662(11) Å) compared to those seen in 79-Ln, which is attributed to the presence of the puckered 18-crown-6 ligand.^115^ The P–Ln–P angles in 82-Ln deviate from linearity, with angles of 161.65(7)° (82-Sm), 154.80(11)° (82-Eu) and 173.98(4)° (82-Yb), again due to the puckered macrocyclic ligand.^115^ As for 81-Yb, the ^13^C{^1^H} NMR spectrum of 82-Yb exhibits virtual triplets for the silyl groups due to coupling with the ^31^P nuclei (*δ*_C_ = 8.43 ppm, ^2^*J*_PC_ = 5.6 Hz),^115^ and higher order effects were also seen in the ^29^Si DEPT90 NMR spectra of 82-Yb (*δ*_Si_ =1.98 ppm, ^1^*J*_PSi_ = 17 Hz), manifesting as virtual triplets.^115^ The ^31^P{^1^H} NMR spectra of 82-Yb exhibits a resonance at −265.58 ppm, with satellites to ^171^Yb nuclei with a ^1^*J*_YbP_ coupling constant of 977 Hz. Similarly to 81-Yb, the ^171^Yb{^1^H} NMR spectra of 82-Yb exhibits the expected triplet resonance at 176.88 ppm.^115^

#### 2.4.2 Actinides

**Fig. 24:**
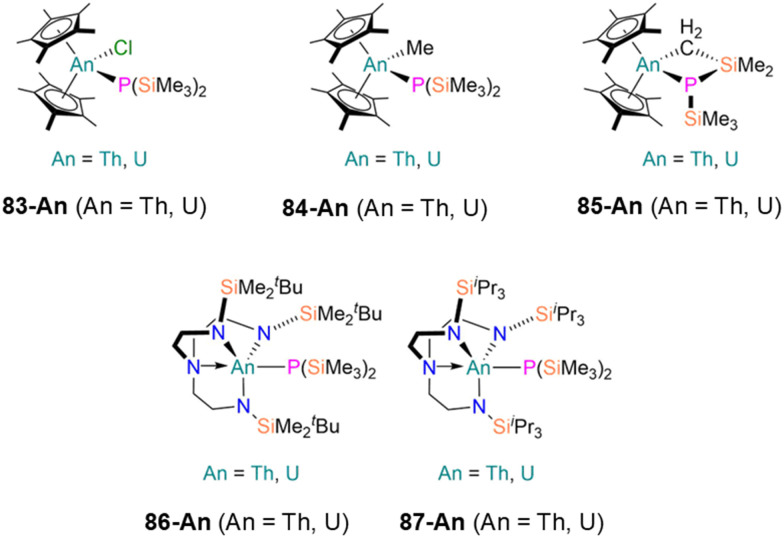
An P′′ complexes [An(P′′)(Cp*)_2_(Cl)] (83-An; An = Th, U), [An(P′′)(Cp*)_2_(Me)] (84-An; An = Th, U), [An{P(SiMe_3_)(SiMe_2_CH_2_)}(Cp*)_2_] (85-An; An = Th, U), [An(Tren^DMBS^)(P′′)] (86-An; An = Th, U), [An(Tren^TIPS^)(P′′)] (87-An; An = Th, U). Tren^DMBS^ = {N(CH_2_CH_2_NSiMe_2_^*t*^Bu)_3_}, Tren^TIPS^ = {N(CH_2_CH_2_NSi^i^Pr_3_)_3_}.

In 1993, Hall reported the actinide (An) P′′ complexes, [An(P′′)(Cp*)_2_(Cl)] (83-An) and [An(P′′)(Cp*)_2_(Me)] (84-An), together with the cyclometallated complexes [An{P(SiMe_3_)(SiMe_2_CH_2_)}(Cp*)_2_] (85-An; An = Th, U); 83-Th and 85-Th were not structurally characterised, Fig. 24.^116^ Complexes 83-An and 84-An were synthesised by the salt metathesis reactions of the respective parent halide complexes [An(Cp*)_2_(Cl)_2_] and [An(Cp*)_2_(Me)(Cl)] with KP′′, though 83-Th may also be prepared from LiP′′.^150^ These complexes showed high thermal stability in the solid state, and solutions of 83-An were also robust towards thermal decomposition. Conversely, solutions of 84-An heated at 393 K for 3 hours led to formation of the metallacycle complexes, 85-An, *via* the elimination of methane; analogous reactivity was previously observed for [U(N′′)_3_(H)].^151^ The U–P bond length for the cyclometallate complex 85-U (2.655(6) Å) was found to be shorter than those of 83-U (2.788(4) Å) and 84-U (2.893(4) Å), whilst the Th–P bond length in 84-Th (2.888(4) Å) is shorter than that seen for 84-U.^116^ In the solid-state structures of 83-U and 84-Th the phosphorus atoms are approximately planar, with angles summing to 357.7(2)° and 355.2(3)°, respectively.^116^^1^H NMR spectroscopy revealed restricted rotation about the An–P bonds of both 83-An and 84-An, with two resonances observed for the inequivalent trimethylsilyl groups that do not coalesce up to 393 K for 83-An.^116^ This restricted rotation was attributed to steric buttressing and was in accord with these complexes having limited further reactivity, including them showing no reaction with KP′′.^116^

In 2018, Liddle, Scheer and co-workers reported the synthesis of a series of An(iv) pnictogen complexes containing bulky triamidoamine ancillary ligands, including the P′′ complexes [An(Tren^DMBS^)(P′′)] (86-An) and [An(Tren^TIPS^)(P′′)] (87-An) (An = Th, U) (Tren^DMBS^ = {N(CH_2_CH_2_NSiMe_2_^*t*^Bu)_3_}, Tren^TIPS^ = {N(CH_2_CH_2_NSi^i^Pr_3_)_3_}, Fig. 24) by the reaction of the respective triamidoamine precursor complexes [An(Tren^R^)(L)][BPh_4_] (An = Th, L = DME; U, L = THF) with one equivalent of KP′′.^117^ It was noted that for 86-Th the Th–P bond length (2.9406(11) Å) was 0.08 Å longer than the U–P bond in 86-U (2.8646(14) Å) despite the single bond covalent radius of Th only being 0.05 Å larger than U.^90^ Conversely, the U–P bond distance in 87-U (2.8391(9) Å) is shorter than that seen for 87-U, and the geometry about the P atom is more planar in the former complex (359.94(5)°) than the latter (354.06(8)°); this was determined by the sum of the angles about the phosphorus atom and the deviation of this value from 360°, which can be attributed to the more sterically demanding Tren^TIPS^ ligand.^117^ As part of a study to probe the propensity of An–E (An = Th, U; E = P, As, Sb, Bi) bonds to undergo either homolytic cleavage or acid–base/dehydrocoupling to occur, 86-U and 87-U were heated to 80 °C and exposed to a 125 W UV lamp for 2 h. It was noted that both complexes are remarkably robust, and undergo <5% thermal and photolytic decomposition under these conditions, likely by homolytic cleavage, whereas acid–base/dehydrocoupling is the most likely decomposition route for more redox-robust Th(iv)–E bonds.

## 3. Bis(triisopropylsilyl)phosphide (P^††^) complexes

In accord with the paucity of structurally authenticated P′′ complexes compared to N′′ across the periodic table, there are even fewer examples of complexes containing bulkier bis(trialkylsilyl)phosphides.^146,152–160^ As bulkier bis(trialkylsilyl)amides such as bis(triisopropylsilyl)amide ({N(Si^i^Pr_3_)_2_}, N^††^) are now starting to grow in popularity as ligands in low-coordinate s-block and f-block chemistry,^5,161^ we also cover bis(triisopropylsilyl)phosphide ({P(Si^i^Pr_3_)_2_}, P^††^) chemistry here; only seven structurally authenticated P^††^ complexes have been reported to date.

**Fig. 25:**
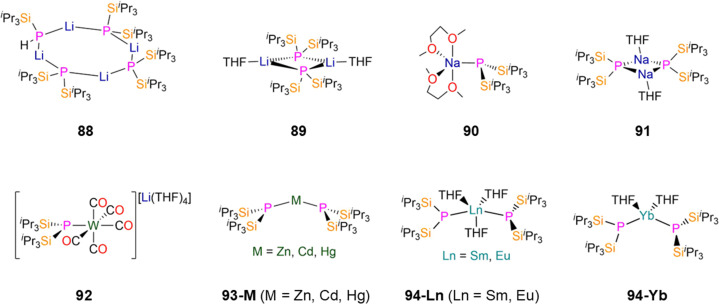
P^††^ complexes [{Li(μ-P^††^)}_3_{Li[μ-P(Si^i^Pr_3_)H]}] (88), [Li(μ-P^††^)(THF)]_2_ (89), [Na(P^††^)(DME)_2_] (90), [Na(μ-P^††^)(THF)]_2_ (91), [W(P^††^)(CO)_5_][Li(THF)_4_] (92), [M(P^††^)_2_] (93-M; M = Zn, Cd, Hg), [Ln(P^††^)_2_(THF)_3_] (94-Ln; Ln = Sm, Eu), [Yb(P^††^)_2_(THF)_2_] (94-Yb).

In 1996, Driess reported the synthesis of [{Li(μ-P^††^)}_3_{Li[μ-P(Si^i^Pr_3_)H]}] (88) by the lithiation of HP(Si^i^Pr_3_)_2_ with ^*n*^BuLi in toluene.^155^ Complex 88 is an eight-membered ring consisting of four Li centres, three bridging P^††^ and one bridging HP(Si^i^Pr_3_) ligand.^155^ Each Li atom in 88 is two-coordinate and the mean Li–P bond length is 2.44(5) Å which is shorter than those seen for the ladder-like Li–P′′ complexes 3 and 4; bulky P^††^ supresses the rearrangement to a ladder-like conformation. The ^31^P NMR spectrum of a solution of 88 in *d*_8_-toluene at −70 °C exhibited two singlets at −338 and −370 ppm and a broad doublet at −351 ppm (^1^*J*_PH_ = 170 Hz). This provided evidence that in solution 88 is a mixed aggregate made up of various [Li{P(Si^i^Pr_3_)_2_}(H)] and [Li(P^††^)] building blocks.^155^ In 2004, von Haenisch published the solid-state structure of [Li(μ-P^††^)(THF)]_2_ (89), Fig. 25, *via* a CSD communication.^162^ Later, in 2005, Westerhausen also reported the synthesis of 89 by analogous methods to that of 88, but with THF used as the solvent.^163^ Complex 89 is a dimeric complex consisting of two three-coordinate Li centres bridged by two P^††^, with each Li bound by one THF.^163^ The Li–P bond length in 89 is 2.533(6) Å, which falls within the range observed for Li–P′′ complexes discussed in this review. It is noted however that although this bond length is neither longer nor shorter than for Li–P′′ complexes, the coordination number of Li in 89 is only three, compared to four for 1.^163^ The ^29^Si{^1^H} NMR spectrum of 89 shows a single resonance at 21.6 ppm for the Si^i^Pr_3_ groups, which exhibits coupling to the ^31^P nuclei (^1^*J*_PSi_ = 49.7 Hz), and one resonance was observed in the ^31^P{^1^H} NMR spectrum at −374.7 ppm.^163^ In 2024, Kays and Mills separately reported the synthesis of NaP^††^ by the addition of two or three equivalents of Si^i^Pr_3_Cl to a refluxing DME solution of red phosphorus, Na metal and naphthalene to yield crystals of either [Na(P^††^)(DME)_2_] (90), or [Na(μ-P^††^)(THF)]_2_ (91) following treatment with THF, Fig. 25.^164,165^ In contrast to P′′ chemistry, where Na salts are yet to be structurally authenticated (see above), the solid-state structures of both 90 and 91 were determined by single crystal XRD. Complex 90 consists of a five-coordinate Na centre with one P^††^ and two bound DME solvent molecules,^164^ whilst 91 is dimeric with a Na_2_P_2_ core and bridging P^††^, with the Na coordination spheres completed by a single THF each.^165^ The mean Na–P bond length in 90 is 2.824(3) Å,^164^ compared to 2.805(2) Å for 91,^165^ which fall within the mean bond lengths discussed for both Li–P′′ and K–P′′ complexes. The geometry of the phosphorus centre in 90 is trigonal pyramidal, with the sum of angles about the phosphorus centre adding to 332.53(10)°.^164^ The ^29^Si DEPT90 NMR spectrum of 90 or 91 in *d*_8_-THF contains a doublet at 20.65 ppm with ^1^*J*_PSi_ = 60.2 Hz, whilst a singlet is observed in the ^31^P{^1^H} NMR spectrum at −384.26 ppm with satellites observed with ^1^*J*_PSi_ = 59.8 Hz.^164,165^

In 2005, Westerhausen reported the synthesis of a W(0) P^††^ complex by addition of a THF solution of [W(CO)_5_(THF)] to 89 to yield [W(P^††^)(CO)_5_][Li(THF)_4_] (92), Fig. 25.^163^ Complex 92 exhibits a distorted octahedral geometry with five coordinated CO and one P^††^.^163^ The W–P bond length of 92 is 2.6665(7) Å and the geometry about the phosphorus centre is trigonal pyramidal (350.38(6)°), which was noted to reflect the shift in the *trans*-CO stretching vibration to smaller wavenumbers (1904 cm^−1^).^163^ As phosphides are strong σ-donors but weak π-acceptors, the π-backbonding from the W to the *trans*-CO ligand is increased, which weakens this W–C bond.^163^ The ^29^Si{^1^H} NMR spectrum of 92 exhibits a triplet at 21.1 ppm with coupling to ^31^P of ^1^*J*_PSi_ = 40.8 Hz, and a singlet resonance at −409.2 ppm is observed in its ^31^P{^1^H} NMR spectrum.^163^ In 2024, Kays reported the synthesis of the group 12 M(ii) complexes [M(P^††^)_2_] (93-M; M = Zn, Cd, Hg) by the salt metathesis reactions of parent ZnCl_2_, CdI_2_ or HgBr_2_ with two equivalents of 90.^165^ The average M–P bond lengths of 2.2263(5) (93-Zn), 2.4215(10) (93-Cd) and 2.3938(7) (93-Hg) Å are consistent with those seen for the terminal P′′ ligands in 68 and 69-M.^83,165^ In addition, complexes 93-M exhibit an increasing P–M–P angle from Zn to Hg (93-Zn; 168.747(12)°, 93-Cd; 169.215(19)°, 93-Hg; 170.086(16)°).^165^ The ^31^P{^1^H} NMR spectra for 93-M exhibit singlets at −287.8, −284.0 and −209.2 for the Zn, Cd and Hg complexes respectively.^165^ For 93-Cd, the ^113^Cd NMR spectrum exhibited a triplet resonance at 137.68 ppm (^1^*J*_CdP_ = 350 Hz, 12.2% abundant ^113^Cd *I* = ½) whereas the ^199^Hg NMR spectrum of 93-Hg contains a triplet at 13.2 ppm (^1^*J*_HgP_ = 407.7 Hz, 16.9% abundant ^199^Hg *I* = ½).^165^ Interestingly, the ^29^Si NMR spectra for 93-Zn and 93-Cd display apparent doublets, whereas for 93-Hg the ^29^Si NMR spectrum is consistent with an AA′XX′ spin system with virtual coupling. This spectrum was accurately simulated using ^1^*J*_PSi_ = 50.6 Hz, ^3^*J*_P′Si_ = 0.0 Hz and ^2^*J*_PP′_ = 19.0 Hz.^165^

In 2024, Mills and co-workers reported the synthesis of a series of three Ln(ii) P^††^ complexes by the addition of two equivalents of 90 to [LnI_2_(THF)_2_] (Ln = Sm, Eu, Yb) to yield [Ln(P^††^)_2_(THF)_*x*_] (Ln = Sm, *x* = 3, 94-Sm; Eu, *x* = 3, 94-Eu; Yb, *x* = 2, 94-Yb), Fig. 25.^164^ Complexes 94-Sm and 94-Eu exhibit five-coordinate Ln centres which are bound by two P^††^ and three THF, whereas 94-Yb features four-coordinate Yb bound by two P^††^ and two THF.^164^ The mean Ln–P bond lengths of 3.0336(13) (94-Sm), 3.0237(18) (94-Eu) and 2.8065(13) (94-Yb) Å are shorter than those seen for the Ln(ii) P′′ complexes 79-M, 81-M and 82-M despite the increased bulk of the P^††^ ligand, though the coordination numbers of 94-Ln are lower.^164^ Complexes 94-M exhibit bent geometries, as evidenced by the P–Ln–P angles (156.11(3)°, 94-Sm; 156.10(3)°, 94-Eu; 133.48(3)°, 94-Yb).^164^ For the diamagnetic complex 94-Yb, a virtual triplet is observed in the ^29^Si DEPT90 NMR spectrum at 24.30 ppm, with coupling to ^31^P nuclei ^1^*J*_PSi_ = 15.8 Hz. The ^31^P{^1^H} NMR spectrum contains a singlet at −301.10 ppm which shows satellites from coupling to ^171^Yb, with ^1^*J*_YbP_ = 1382.1 Hz, as well as to ^29^Si, ^1^*J*_PSi_ = 18.1 Hz.^164^ The ^171^Yb{^1^H} NMR spectrum of 94-Yb contains a triplet resonance at 682 ppm due to coupling to two equivalent ^31^P nuclei (^1^*J*_YbP_ = 1382.9 Hz).

## 4. Conclusions

We have shown that metal P′′ coordination chemistry lags far behind that of its lighter congener N′′, and that there are therefore a huge number of avenues to be explored and exploited. Although the CSD search performed above gave 210 P′′ *vs*. 4062 N′′ metal complexes, we note that an analogous search for the other heavy group 15 congeners As′′ (101), Sb′′ (58) and Bi′′ (22) returns progressively fewer hits upon descent of the group.^9^ Perhaps the most surprising omissions in s-block metal P′′ chemistry are that there are no structurally characterised examples of Na or Be P′′ complexes to date.^9^ The former of these absences is quite remarkable considering that synthetic routes to Na P′′ salts are well-known,^12^ and that solid-state structures of solvated Na P^††^ complexes are already known despite the chemistry of this bulkier bis(silyl)phosphide ligand being in its relative infancy.^164,165^ We have found there to be relatively few p-block metal P′′ complexes, including no structurally authenticated group 15 and 16 metal(loid) P′′ complexes to date.^9^ This is quite astonishing considering the potential of such molecular precursors in the synthesis of solid-state III–V semiconductors;^42–57^ we noted that a Bi P′′ complex has been reported without a solid-state structure,^12*e*^ and this is also the case for Sb.^166^ We have identified multiple gaps in d-block metal P′′ chemistry that can be exploited, mainly for the second and third row d-transition metals. There are ten d-transition metal M–P bonds that have yet to be structurally authenticated for this ligand, surprisingly including the entirety of groups 5 and 9.^9^ We have discussed that numerous 18 e^−^ Fe(ii), Ru(ii) and Os(ii) P′′ complexes have been synthesised but not yet structurally characterised;^99^ similarly, there are numerous other 18 e^−^ d-transition metal P′′ complexes that are known but lack solid-state structural data, including Mn(i)^167^ and Re(i)^168^ examples. The synthesis of group 7 M(i) and group 8 M(ii) P′′ complexes are particularly promising avenues to pursue as these are diamagnetic and follow the 18 e^−^ rule, allowing reactions to be easily monitored by ^31^P NMR spectroscopy and in principle providing relatively stable products.^22,23^ Finally, there is also a notable paucity of f-block metal P′′ complexes, considering how crucial the N′′ ligand has been to the development of molecular f-block chemistry,^5^ and this has only started to be addressed relatively recently.^115,116,164^

The structural data discussed herein has showcased how the flexibility of coordination modes and geometries of P′′ provides rich coordination chemistry that juxtaposes its lighter congener N′′ and other bulky bis(silyl)phosphide ligands such as P^††^ and {P(SiPh_3_)_2_}.^12*e*,146,169^ The softer nature of P′′ *vs*. N′′ could be exploited in the stabilisation of low oxidation state metal complexes and unusual structural motifs, and the greater tendency of P′′ to bridge metals and form oligomers can be harnessed to provide multimetallic complexes that can be used as precursors for catalysis, superconductors and magnetic materials. With the exception of s-block P′′ complexes, which have been widely applied as ligand transfer and reducing agents,^12,18–20^ we note that relatively few reactivity studies have been reported for other structurally characterised p-, d- and f-block metal P′′ complexes to date. This is an unusual observation given the rich chemistry of metal N′′ complexes across the periodic table,^1–7^ and that both insertion of unsaturated substrates into M–P bonds and cleavage of P–Si bonds are relatively facile processes for P′′ complexes.^15–23,99^ We envisage that future investigations of P′′ coordination chemistry, as well as its heavier As, Sb and Bi congeners and bulkier derivatives such as P^††^ and {P(SiPh_3_)_2_} in tandem, will provide examples that complement and contrast to those of better-understood N′′ complexes.

## Data availability

No primary research results, software or code has been included and no new data were generated or analysed as part of this review.

## Conflicts of interest

There are no conflicts to declare.
